# Adolescent girls’ explanations of high rates of low mood and anxiety in their population: a co-produced qualitative study

**DOI:** 10.1186/s12905-024-03517-x

**Published:** 2025-02-04

**Authors:** Ola Demkowicz, Rebecca Jefferson, Pratyasha Nanda, Lucy Foulkes, Jo Lam, Steven Pryjmachuk, Rhiannon Evans, Bernadka Dubicka, Liz Neill, Laura Anne Winter, Georgina Nnamani

**Affiliations:** 1 Manchester Institute of Education, The University of Manchester, Manchester, UK; 2 Common Room, Leeds, UK; 3 Department of Experimental Psychology, University of Oxford, Oxford, UK; 4 Division of Nursing, Midwifery, and Social Work, The University of Manchester, Manchester, UK; 5 DECIPHer (Centre for Development, Evaluation, Complexity and Implementation in Public Health Improvement), School of Social Sciences, Cardiff University, Cardiff, UK; 6 Division of Neuroscience, The University of Manchester, Manchester, UK; 7 Hull and York Medical School, University of York, York, UK

**Keywords:** Gender, Adolescent girls, Mental health, Low mood, Anxiety, Qualitative, Co-production

## Abstract

**Background:**

From early adolescence, girls face greater risk of experiencing low mood and anxiety relative to boys, with recent evidence that this may be worsening. There is a paucity of mental health research that meaningfully progresses understanding of these gender disparities, including that engages adolescent girls’ own perspectives, limiting our ability to direct further research and enhance intervention approaches.

**Aims:**

We examined low mood and anxiety from the perspective of adolescent girls, asking: *What do adolescent girls perceive to be causing their population’s high rates of low mood and anxiety?*

**Methods:**

We adopted a co-produced qualitative design, guided by ecological systems theory, conducting focus groups in 2022 with 32 adolescent girls aged 16 to 18 years in England. Data were analysed using reflexive thematic analysis.

**Analysis:**

Participants framed low mood and anxiety among adolescent girls as “normal”, and discussed potential explanations including persistent reiteration and expectation of gendered norms, intense educational pressures in ways that can be gendered, difficulties within peer relationships, and comparison and insecurity in social media contexts. Throughout, participants highlighted how complex these issues are, including nuances around individual differences, sociodemographic contexts, and societal contexts.

**Conclusions:**

The study offers a critically important contribution to evidence on gendered inequalities in low mood and anxiety, drawing attention to the interwoven and complex nature of girls’ lives and illuminating various aspects that would benefit from greater research. The insights gained through exploration with girls themselves hold policy and practical relevance to enhance systems to meet girls’ needs.

**Supplementary Information:**

The online version contains supplementary material available at 10.1186/s12905-024-03517-x.

From early adolescence, girls experience greater risk of low mood and anxiety relative to boys, and from mid-adolescence onwards are twice as likely to report depression [1–3]. In recent years, evidence suggests that these rates have increased among adolescent girls [4–7], and the COVID-19 pandemic appeared to exacerbate this [8, 9]. Such evidence constitutes a growing public health concern warranting investigation [10, 11], particularly given a longstanding paucity of research meaningfully unpacking the gender mental health gap [12]. We note comparisons among girls/women and boys/men partially because much established evidence lacks recognition of transgender and nonbinary adolescents, though evidence suggests that they are also more likely to experience poorer mental health [13]. We use ‘girls and women’ inclusively, but acknowledge that the literature drawn upon often uses sex-based language and sampling.

Research and policy highlights uniqueness in the drivers and patterns of mental health among girls and women. For instance, it has been suggested that girls may face distinct risk factors, elevated prevalence of risk factors, or be differently impacted by risk factors relative to boys [14] and that there may be underlying vulnerabilities that interact including to heighten the impact of risk factors [14–16]. This necessitates gender-sensitive preventative research, action, and provision [17], and particularly attention to the adolescent period where gendered patterns in low mood and anxiety seem to emerge and widen. Besides possible pandemic effects [8, 9], causes of increased low mood and anxiety among girls remain unclear. Researchers have posited *possible* contributing factors spanning various life domains, which echoes ecological systems theory’s framing of health and development as occurring in the context of multiple transactional environments and processes [18]. These potential factors include social media usage, increased sexualisation of adolescent girls, increased academic pressure, and limited school provision available in relation to low mood and anxiety despite already heightened prevalence among girls [4, 5, 19]. There have also been considerable societal shifts in mental health discourses (i.e., an uptick in mental health awareness campaigns, school-based mental health education, and an increased use of mental health language in day-to-day discourses with adolescents), which have been theorised as potentially increasing willingness to report [20, 21].

There is some theoretical and empirical merit for the above factors as potentially explaining the increase in rates. However, understanding of how these factors link to adolescent girls’ mental health specifically, and how they explain current rates of low mood and anxiety, is still developing, and research is limited in various ways. For instance, relating to the example of sexualisation, some emerging evidence indicates that perceived sexualisation and objectification [22] and sexual violence experiences in mid-adolescence [23] are associated with low mood and anxiety among girls. This includes some longitudinal UK-based evidence [23], though the evidence base as a whole faces critical limitations in the level of detail in available data (e.g., with little information available on recency, severity, frequency), limiting our ability to understand nuance in a complex experience. Evidence also suggests that girls may experience greater anxiety around specific educational pressures (e.g., cross-sectional evidence on maths anxiety [24]), and a qualitative systematic review explored how gendered “imbalances” in education pressures (e.g., over-emphasis on achievement, gendered expectations, heightened personal pressure) appear intertwined with worsened mental health [25], though evidence in this area remains sparse, mixed, and largely cross-sectional [26].

As such, previously suggested explanations for increased rates of low mood and anxiety among girls require further investigation to consolidate and extend this evidence base. Importantly, explanations have come from researchers without clear input from adolescent girls, limiting capacity to meaningfully explore and address inequalities. From a complex systems perspective – where real-world systems are viewed as intricate, dynamic, and context-dependent – intervention development should begin with a deep understanding of how the target population perceives both determinants and needs [27]. Investigation of girls’ and women’s own perspectives is, however, frequently overlooked in women’s health issues [12, 17]. There is, therefore, a need to engage with adolescent girls themselves to understand their perspectives on rates of low mood and anxiety.

Several qualitative studies have engaged adolescent girls on specific issues including some factors explored above (e.g., what ‘problematic’ versus ‘healthy’ social media engagement might mean for wellbeing dimensions such as body image) [28, 29], though few studies have explored adolescent girls’ understandings of how gendered experiences contribute to mental health including low mood and anxiety. Engaging adolescent girls in discussions may highlight issues including those not anticipated by researchers, and inform ongoing research, policy, and practice priorities. Some studies have used such an approach, highlighting challenges for adolescent girls that appear linked to longstanding mental health disparities. This has commonly included considerable daily pressure and responsibility, and sexual harassment and violence and related fear and restricted freedoms [30–34]. Some highlighted competing societal femininity discourses, including traditional gendered norms, feminist ideals, and male-dominated ideals, that are difficult to assimilate and thus experienced as upsetting and worrying [31, 32].

However, data in the above qualitative studies were generated over ten years ago, so cannot offer insights around subsequent increased rates of low mood and anxiety observed since. Most were conducted in Sweden, the fourth most gender-equal country according to the 2021 Gender Inequality Index [35]. It is likely that national and local contexts (e.g., in education systems, economic contexts, relevant laws, cultural and religious influences) may influence both gender and mental health experiences [36], and exploration in varied contexts can therefore be locally insightful *and*, when considering studies collectively, offer transferable global implications. In the UK (27th in the 2021 Gender Inequality Index [35]), a very small number of studies have produced some insights through asking adolescent girls about wellbeing-related factors, but lacked specific detail regarding low mood and anxiety. For instance, The Children’s Society’s Good Childhood Report [37] explored gender differences in subjective wellbeing, and shared some adolescent girls’ comments around attractiveness expectations, but did not offer in-depth analysis or examine mental health components including low mood and anxiety, limiting specificity in inferences.

## Aims

We examined the factors potentially related to the high rates of low mood and anxiety from the perspective of adolescent girls themselves, asking: *What do adolescent girls perceive to be causing their population’s high rates of low mood and anxiety?* We were guided theoretically by ecological systems theory [18] given that researchers’ hypotheses for current rates of low mood and anxiety span ecological domains, and as use of this theory in qualitative research enables recognition of systemic inequality processes [38].

## Methods

### Design

We adopted a qualitative design, conducting online focus groups in May–August 2022 with adolescent girls aged 16 to 18 years in England. The project was co-produced with young researchers (Authors PN and JL) to embed young people’s perspectives throughout project design, implementation, and interpretation, embedding youth voice to facilitate us to better engage with our participants and more meaningfully interpret their experiences. Young researchers were active members in all stages of the design process, including developing recruitment and data generation approaches, co-leading focus groups, analysing data, and contributing to dissemination. More detailed reflections on co-production are shared in the Supplementary Materials. We pre-registered the study (Research Registry ID: researchregistry7803) and agreed a protocol prior to recruitment (publicly available alongside data generation documents using Open Science Framework; 10.17605/OSF.IO/KBTA4).

### Research team

Our team includes research academics, clinical academics, young people, and youth engagement specialists. Most of us are women, and some of us are from UK ethnic minority and LGBTQIA+ communities. Several team members bring lived experience of mental health difficulties. We share interests in adolescent mental health, with expertise around gendered experiences, time trends, public health, and social justice. Some bring clinical backgrounds (Authors SP, BD, LAW), and young researchers (Authors PN, JL) have experience working in other research projects and in training and provision in child and youth mental health services.

### Participants and sampling

Our target population was diverse adolescent girls aged 16 to 18 years across England. Low mood and anxiety rates are highest in this older age group [7], and we anticipated that they could likely draw on a fuller range of experience than younger adolescents and possess greater critical understanding around complex issues [39]. We sought ‘diverse’ girls as gendered experiences intersect with other identities such as ethnicity, class, and sexual orientation [40]. We did not set specific targets for numbers of participants from particular groups; rather, we aimed to approach this thoughtfully, recognizing the importance of recruiting a sample with varied contexts.

We interviewed 32 participants across eight focus groups, with 3–5 participants in each group (mean group: 4). We aimed for 40–45 participants, with 5–6 individuals in each group, as is moderate within focus group research [41]. However, recruitment and data generation timing (alongside exams and the final stages of educational engagement for Summer) meant lower responsiveness from gatekeeper organisations and, in turn, adolescents. Qualitative sample sizes should be guided by features such as the dataset’s richness and detail [42], and we were also working to include diverse voices; given the depth of participants’ contributions and our sample’s diversity, we determined that 32 participants was more than adequate.

We recruited via post-16 education settings (i.e., settings that provide educational opportunities to students aged 16 and above, including in England sixth forms, colleges, and vocational training centres) and youth organisations (e.g., charities, youth clubs) in England, contacting key gatekeepers for these organisations and advertising via networks (e.g., Anna Freud Schools Division, National College Association). Members of the research team asked interested organisations to share an advert, which included illustrations of girls who varied in their appearance to encourage girls from diverse backgrounds to take part (e.g., with varied expressions of gender and ethnicity), with 5–10 adolescent girls. Interested participants expressed interest and provided demographic information to us via an online form; we intended to use this information to strategically identify a diverse sample. However, given slow recruitment, we invited all interested individuals.

Participants were 16 (*n* = 6), 17 (*n* = 24), or 18 (*n* = 2) years old. Of 32 participants, 15 identified themselves as white, 9 as Asian, 4 as Black, 2 as mixed, and 2 as another ethnicity. In terms of sexual orientation, 20 participants indicated that they were heterosexual, 5 as bisexual, 2 as gay or lesbian, 1 as queer, 1 as asexual, 1 as not sure, and 2 preferred not to say. For religion, 14 identified as having no religion, 7 as Christian, 6 as Muslim, 2 as Hindu, and the remaining 3 preferred not to say. Half (*n* = 16) indicated having sought help from mental health services, which we defined for participants as a wide range of available services including, for example, GPs, school counsellors, and psychiatric services. Only 2 participants shared having been eligible for free school meals, and 2 shared having special educational needs. Participants were from most regions of England, with 11 from the North West, 10 from the South East, 3 from the East Midlands, 2 from the West Midlands, 2 from London, 3 across the East of England, South West, and Yorkshire, respectively, and 1 who preferred not to say.

### Data generation

We used focus groups given our focus on perceptions rather than direct personal experience, which can benefit from contrast and clarification of views within groups [43]. One university researcher (RJ) and one young co-researcher (PN, JL) co-led sessions to minimise power differentials, facilitate open, democratic discussions, and draw out insights from a youth perspective that could help the young researchers to unpack participants’ answers [44]; we provided training for young researchers in advance.

We brought together participants who were unknown to each other, rather than existing peer groups, as prior dynamics and shared understanding can influence discussions [43]. We conducted groups online, to limit existing relationships among participants, reduce burden on young co-researchers, and lower time and budget, and used Zoom given advantages of cost-effectiveness, user-friendliness, security, and flexibility for varied needs [45]. Sessions lasted 90 min, including introductions, ground rules, and debriefing, consistent with guidance on good practice in focus groups [43], with discussion time ranging 63–78 min. Focus groups were audio-recorded and transcribed verbatim.

We followed a semi-structured guide (see Table 1) using open-ended questions and follow-up prompts to explore answers, including mechanisms and group variations. We explicitly introduced the concept and evidence of high rates among girls to begin discussions, and then created space for open discussion of participants’ perspectives, introducing ecological system domains, guided by ecological systems theory [18], to elicit varied insights rather than asking about narrow pre-determined experiences (see prompts 3c/3d. We established ground rules (e.g., listening respectfully, maintaining confidentiality) and managed social dynamics (e.g., dominant/quieter participants).

**Table 1:** Focus group questions and prompts used

1. Firstly, what do you think when you hear that teenage girls have been reporting greater levels of low mood and anxiety?
a. What makes you think this?
2. Does this feel true to the experiences of you and your peers during your teenage years? By peers we mean the other girls you have known during your teenage years, such as your classmates.
a. Could you expand on this?/What makes you think this?
3. We want to know what you think might be contributing to that. To start us off, what do you think the top three things might be?
a. Can you tell us a little more about that?
b. How do you think this might contribute specifically to low mood and anxiety for girls in recent years?
c. Do you think there is anything else in day-to-day life that might be contributing to low mood and anxiety for girls? *Examples of domains if needed to help prompt discussion: family and home; school; social relationships with friends, peers, or romantic relationships; digital platforms/interaction and social media; considerations about the future*
d. What about issues at a wider society level? *Examples if need to help prompt discussion and clarify what we mean: how we talk about girls and women, systems such as the law.*

### Ethical considerations

The project received approval from The University of Manchester Research Ethics Committee (Ref: 2022-13633-23090). We established informed consent, providing written information alongside a video with group moderators (RJ, PN, JL), ensuring that participants received this information at least one week before a focus group. Grouping participants without existing relationships minimised confidentiality risks, and we established ground rules not to share personal information, gave instructions for displaying first names only, and set meeting passwords. We stored data on secure University systems and pseudonymised accounts. We limited potential distress by asking participants not to share anything deeply personal or upsetting and advising that individuals who may find this topic upsetting should not take part, and providing signposting.

### Data analysis strategy

We used Braun and Clarke’s reflexive thematic analysis (RTA) [46, 47], wherein researchers construct a thematic representation of dataset patterns and nuances. Given our exploratory design and emphasis on adolescent girls’ perspectives, we analysed inductively rather than through a theoretical framework. That is, we used ecological systems theory [18] to shape the scope of data generation questioning and interpret findings post-analysis, rather than actively incorporating this into analysis. We used semantic coding, focusing broadly on explicit statements rather than possible underlying meanings.

We co-produced analysis, undertaken by Authors OD, RJ, PN, and LF, with a training workshop for PN at the outset. Consistent with RTA, our team approach was grounded in building shared reflexive interpretations and not to narrowly establish ‘coder reliability’ [47]. We familiarised ourselves with data, and discussed initial reactions. Authors OD and RJ divided transcripts and systematically coded to identify units relevant to our research question, organised within NVivo Version 12 [48]. Author PN reviewed this coding to expand and develop codes and thus facilitate shared interpretation. Next, analysts met to collaboratively group codes into themes, setting aside some codes (e.g., isolated points, those less directly relevant to the research question). Author OD reviewed and refined themes, regularly debriefing with the team, and consulted Author JL as second young researcher as a sense checking exercise. Codes underpinning themes are in Supplementary Materials for transparency, though we highlight that RTA is an interpretive process and analysis is not a definitive reading, but *one* credible account [44].

## Analysis

**Fig. 1:**
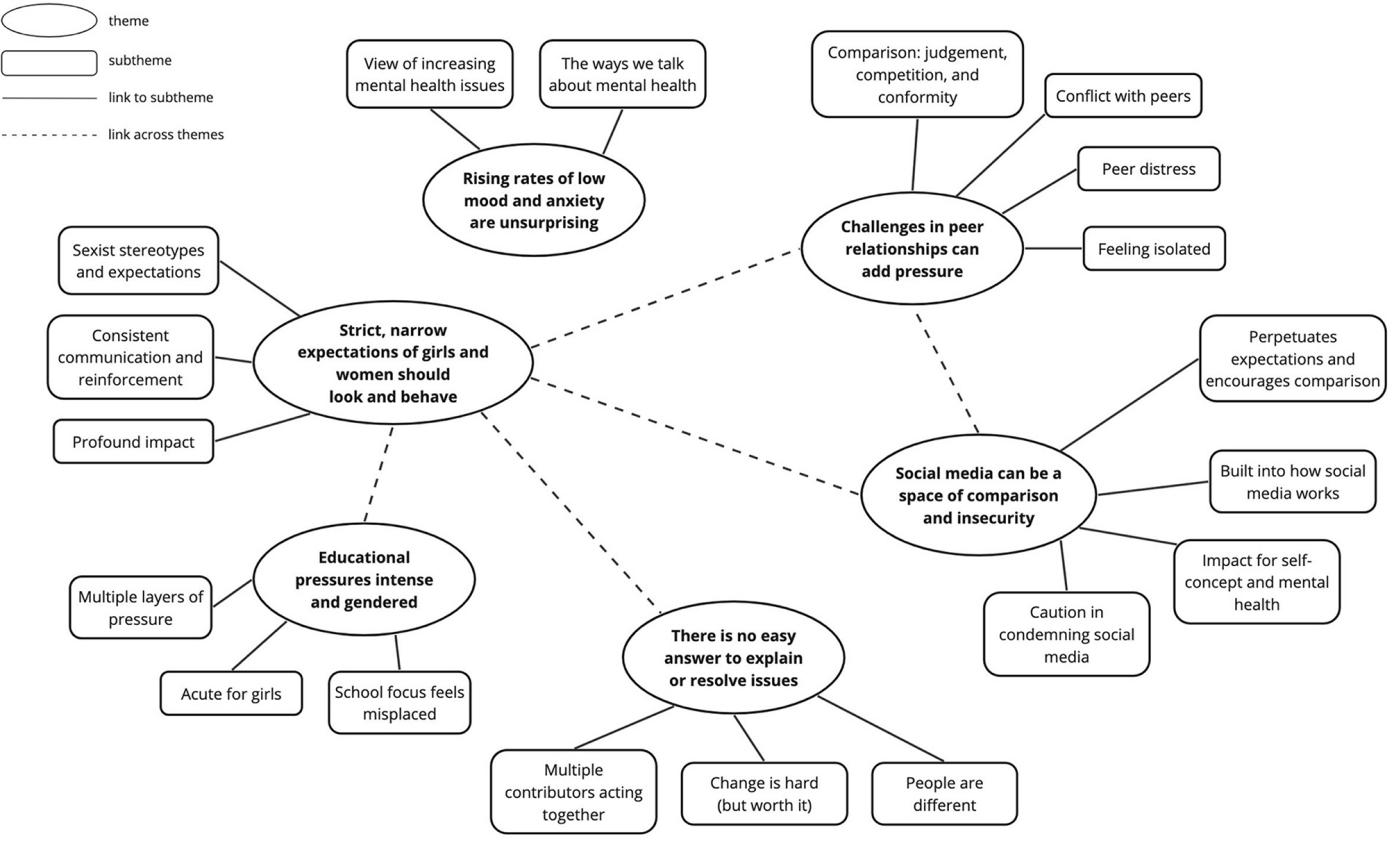
Thematic map presenting six main themes alongside their associated subthemes

We developed six main themes, shown alongside their respective subthemes and connections in Fig. 1. Here we explore these themes, drawing upon illustrative quotes accompanied by pseudonyms to evidence and contextualise, and attending to agreement, nuances, and divergences in participants’ viewpoints.

### Theme 1: Rising rates of low mood and anxiety are unsurprising

We began discussions by introducing evidence of heightened rates of low mood and anxiety among girls relative to boys, including of apparent recent increases. There was consensus that this evidence was unsurprising (though in some instances comments seemed to relate broadly to increases among adolescents, rather than adolescent girls specifically): “it’s not really a shock, it’s just something we all know” (Sophie). Participants said that evidence echoed what they felt to be true, referencing their own experience, their peers’ experiences, and wider messaging: “it doesn’t shock me because there’s a lot of [my friends] that experience it” (Phoebe); “I heard that mental health problems have been increasing” (Katrina). Some expressed that to experience difficulties like low mood and anxiety is normal among adolescents now: “I’ve definitely seen an increase. It’s more of a normal thing for people to go through” (Alex); “I think it is just normal for teenage girls to have low mood” (Isa). Participants pointed to the COVID-19 pandemic as exacerbating issues: “when I think of increasing mental health issues like among teenagers I think of COVID and lockdown and stuff” (Louise).

Participants also described a normalisation of talking about mental health among adolescents, who they saw as increasingly comfortable talking about difficult feelings and mental health challenges: “people are more open about things, so they talk about it” (Amina). However, a few participants suggested that poor mental health is idealised: “in a lot of mental health education mental health is romanticized almost. I feel like some people want to have mental health issues” (Beth), and that normalisation only extends so far: “it’s OK not to be OK, until it gets messy” (Alex).

### Theme 2: Strict and narrow expectations of how girls and women should look and behave

Participants described sexist stereotypes and expectations as being placed on and internalised by adolescent girls, embedded in a longstanding conceptualisation of women as restricted to particular patterns of behaviour and expression: “men are always seen as the more favoured and they’re more able, they’re more capable. I still think people look at women and think, ‘Oh, you shouldn’t do this, you shouldn’t do that’” (Maya). These included overarching perspectives of who girls and women “should” be as well as specific aspects of behaviour and appearance; i.e., that girls should be quiet and polite, and should aim to be beautiful within narrow, homogeneous standards:

Stuff like makeup and shaving has become so normalized it’s no longer a choice that you make, it’s kind of… expected. So, you don’t really get to decide what is beautiful to you, but, you’re just kind of forced to play into it (Emmy).

Participants explored how such expectations are communicated and reinforced from an early age, such as in childhood toys and media: “if you think like the toys boys and girls are given, a girl would get given a kitchen set, being in a kitchen’s quite quiet, but a boy’ll get given a nerf gun, that’s loud” (Ayesha). They highlighted that the media generally portray women in narrow and gendered ways, often with implicit or explicit judgement: “one of the main reasons [for low mood and anxiety] might be the media, because there’s quite a large very focus on women’s appearance and also personality, especially compared to the men” (Katrina).

Participants reflected on how these expectations are reiterated to adolescent girls by peers and adults, with gendered comments and judgement of them when one is not as not conforming somehow: “you’re sort of performing… you have to look a certain way, and smile, or if you don’t smile, you get told to smile” (Ayesha). Some reflected feeling that *all* ways of being are judged for girls, meaning they exist in an impossible space of being:

Abigail: In primary I was the only girl that would play football and I was called a tomboy, the whole of primary literally from year three onwards, and then….

Sunita: Did you ever get told that it was cause you had a crush on the boys?

Abigail: Yeah.

Sunita: ’Cause- [splutters] I hate that so much.

Abigail: Yeah, like, either you’re a tomboy of it’s like ‘oh she just wants to be friends with the boys.’ […]

Kira: And leading on from Sunita’s point where girls are made fun of for whatever they like. Like if you’re girly then you’re made fun for being too girly. But if you’re kind of like a tomboy, you’re made fun of that as well, like you’re a pick me girl or whatever. So, no matter what you’re always going to be judged in some way.

These narratives were explored as permeating and limiting other aspects of day-to-day life for adolescent girls. For example, participants talked about how this permits a normalisation of sexual harassment in adolescent spaces (particularly schools), how options available to them such as sports and STEM subjects (science, technology, engineering, and mathematics) are narrowed, and how girls see coverage of societal issues affecting women being minimised: “when a boy is like pinching a girl or something like that, and like physically doing things they’ll just say it’s flirting, and blame the girl” (Sunita).

This pervasive messaging of gendered expectations, and the ways that narratives around gender and women permeate day-to-day life, was framed as having a profound impact for adolescent girls. Participants explored how expectations become normalised and unconsciously internalised, leading to feelings of insecurity and becoming deeply bound up with one’s identity, self-concept, and self-worth:

When people think that insecure girls is just someone who thinks, ‘oh, my hair isn’t good enough’, but it’s so much deeper than that and I think a lot of the insecurities within girls that they just don’t feel like… a person because of how they’ve been treated (Sunita).

[Society’s conceptualisation of women as inferior] just makes [girls and women] feel like they’re like worthless (Sara).

### Theme 3: Educational pressures feel intense and gendered

Participants described educational experiences as restrictive and pressuring, and not always considerate of developmental, wellbeing, and individual needs. They discussed pressure and demand – from teachers, from parents/carers, and implied within systems – around attainment and behaviour, with no room for mistakes, that become especially intense across secondary school and further education (e.g., sixth form, college) around exams and grades, making major life decisions, and not only keeping up but being “the best”. They reflected that these can lead to persistent feelings of sadness and worry: “in year 9 I was perfectly fine and then in year 10 I was really sad and really depressed and I had so much anxiety because I really wanted to do well in my GCSEs” (Hanna).

Participants explored how educational pressures may be communicated to and experienced by girls in acute, complex ways. Some felt that educational pressure is greater for girls, because high achievement is expected, heightening the emotional impact: “if a boy fails, then, you know, it’s not that bad, but I feel like if a girl does, it means a lot more” (Ayesha). As in Theme 2 (*Strict and narrow expectations of how girls and women should look and behave*), participants reflected on gendered expectations of classroom behaviour and in how their classroom contributions are valued, which could feel upsetting and diminish their sense of self. A particular issue explored here was ‘male-dominated’ STEM subjects, which were seen to be gendered spaces with low expectations, creating additional pressure and worry around achievement: “if you’re in like a male-dominated classroom and you’re getting the worst results, it kind of… you just feel like you take all of that on yourself and you just feel like you’re letting down other women” (Emmy).

### Theme 4: Challenges in peer relationships can add pressure

Challenging aspects of peer relationships were frequently explored as linked to low mood and anxiety. Participants spoke of a culture of comparison among adolescent girls, where it is normalised to compare oneself to others and to judge and feel judged by others, spanning domains including ways of thinking, grades, future life plans, and, most prominently, appearance and self-expression:

It became a competition to see if you could eat the least at lunch basically. So it was a whole table of girls all with tiny little plate of salad on their plate and then if you didn’t join in with that you felt like you were being judged, because you were sat there eating pasta or something, and someone was like ‘oh my gosh that’s so much carbs, you’re going to get fat’ (Beth).

Participants explored such dynamics as partially rooted in gendered societal narratives and insecurity, and that this comparison and competition can be stressful, encouraging of rumination, and problematic for self-esteem and sense of self: “most people are quite insecure and then they feel the need for competition to validate how they feel” (Liah).

Participants also pointed to conflict with peers, including arguments and fallouts between friends and peers, challenges experienced in romantic relationships and breakups, issues of bullying in a peer context, and feeling isolated, all of which seep into online and offline worlds and can prompt considerable distress: “the fallout of breakups and arguments can be quite upsetting. I know when I was in secondary school that was the main reason why I was quite upset because I’d have arguments with friends” (Kate); “when girls are mean it’s less direct […] so you feel a bit crazy” (Katrina).

Some wondered whether low mood and anxiety among girls could become self-perpetuating through friendships, exploring the possibility of emotional contagion among girls, and how challenging it can be to support a friend experiencing such feelings: “if the numbers of young teenage girls who are having bad mental health or just low mood or anxiety then, if they’re going up […] it’s just going to kind of like bounce off each other (Liah). However, participants also described friendships as valuable mental health support sources, with the challenges captured in this theme comprising only part of these connections: “the one thing that will always help me is my best friend” (Sunita).

### Theme 5: Social media can be a space of comparison and insecurity

This theme is interconnected with several wider themes, as we found that the ways that participants discussed social media framed it as a platform in which the other issues they described (e.g., gendered expectations, challenges with peers) play out or are magnified. Participants framed comparison and insecurity as normalised in social media spaces, and emphasised that platforms with more visual content (e.g., Instagram, TikTok) reiterate expectations for how girls should present themselves. Participants explored how this leads to comparison to peers and to public figures including ‘influencers’, and normalises a sense of insecurity and conforming to these expectations: “they’re seeing an image over and over again whether it’s videos on TikTok or Instagram etcetera, so they feel like that’s what they have to look like or else they’re ugly” (Hanna).

Participants noted how the standards established on social media around one’s appearance and day-to-day life are generally unrealistic: “you see these people with perfect lives and the perfect house and a perfect face” (Kira). They highlighted that it can be difficult to tell what is “real” (particularly for younger adolescents), meaning that adolescent girls are often engaging in unreasonable comparisons: “you only see the best bits of people’s lives and sometimes that is really hard to separate out” (Alex). They noted particular ways that platforms function as problematic here, such as normalisation of filters and photoshop, and algorithms that narrow content engagement: “there’s filters that are really realistic, so you wouldn’t know” (Abigail); “if you watch dancing videos or makeup and stuff revolving around ‘body’, then you’ll see more of that […] and then you think ‘maybe that’s how my body should look’” (Isa).

Participants therefore raised concerns about adolescent girls being so engaged with these platforms, including during childhood, and linked this normalisation and comparison to low mood and anxiety among adolescent girls: “in your mind you’re comparing yourself without even thinking about it and that affects your mood” (Kira); “I think [the increase of low mood and anxiety among girls] is because of different social media apps on different social platforms” (Amina).

Yet, participants reflected that it is easy to overly blame rates of mental health difficulties on social media, and advised caution in treating this association as one-dimensional. They explored perceived positives of social media as a platform for body positivity, mental health conversations, queer communities, and connection and community, and suggested that positive and negative aspects are intertwined: “the increasing connectedness that we have with people all over the world is great, but it’s also really bad because you can compare yourself to everyone else, and I think that’s really damaging” (Sophie).

### Theme 6: There is no easy answer to explain or resolve issues

Throughout discussions there was an underlying narrative of the complexity of the issues explored. Some participants reflected upon the idea that there is not any single factor driving rates of low mood and anxiety among adolescent girls, including recent increases: “I think there’s a whole lot of different things causing this trend” (Sophie). Participants unpicked the extent to which one can draw overarching conclusions about what affects adolescent girls and their mental health. They explored how many of the issues being discussed did not only affect adolescent girls, also affecting boys and people at other life stages: “yes, girls have body image issues sometimes because of social media, but sometimes young boys have this image of how a man should look like…. that can really degrade their mental health” (Isa). Some participants emphasised individual differences in experiences, including in exposure to different contributing factors and experiences to one another, in how people respond to similar factors and experiences, and in support needs. This included in relation to intersecting experiences among groups such as those from UK ethnic minority and LGBTQIA + communities: “when it comes to sexuality other people definitely are like the reason for like low mood and bad mental health [because of how those other people respond]” (Ruby).

Participants reflected on how the issues they discussed were complex and systematic (e.g., misogyny, social media norms), rendering change challenging, and requiring those in positions of power to listen and embrace change: “how can you convince millions of people to develop a different mindset [to] change our thinking?” (Rana); “[it would be helpful] if politicians actually talk to people who’ve gone through all of this, is still going through it, like we are now” (Alex). Yet, participants emphasised that this change is important, even if it is hard and requires incremental progress: “even if one little certain bit can be tackled then it’s one certain bit that helps, like, a person” (Chloe).

## Discussion

This study offers important insights into girls’ perspectives on potential contributors to their population’s high rates of low mood and anxiety. We present an account from the adolescent girls who participated that to experience low mood and anxiety as an adolescent girl is “normal”, both generally as part of the adolescent experience but particularly among girls in their generation (though notably, in some instances this seemed a broad comment on what is normal among adolescents generally rather than specifically only for girls). Participants explored the reiteration of gendered norms from early childhood and persistent judgement of girls’ presentation and behaviour, and discussed profound implications for identity, self-esteem, and, consequently, mental health. They reflected on restrictive, pressurising educational experiences (including aspects they felt may be acute and complex for girls), which they noted as creating considerable anxiety. They explored challenges in peer relationships, including comparison and conflict, that can be stressful and prompt low mood and anxiety. Finally, participants framed social media as a place where comparison and insecurity are normalised and where various standards, often unrealistic, are reiterated in ways that can affect mood and create worry. The complexity of such issues spanned discussions, with participants guarding against oversimplifying explanations and overlooking individual differences and sociodemographic contexts, and reflecting on how embedded and systemic many issues are.

Some of the above factors have been previously evidenced as associated with adolescent low mood and anxiety, including to some degree for girls specifically. Quantitative studies have indicated a relationship between gender norm perceptions and low mood and anxiety among adolescent girls [49], and other qualitative studies have similarly suggested gender norms can be stressful and elicit upset and worry among girls [31, 32]. Quantitative and qualitative studies have indicated that educational pressure and gendered educational discourses (i.e., imbalanced expectations that girls be well-rounded high-achievers, as was also noted by our participants in Theme 3, *Educational pressures feel intense and gendered*) may have specific ramifications for girls [25, 50]. There are evidenced complexities around peer relationships and adolescent mental health, with studies indicating that ‘negative’ (e.g., bullying) *and* ‘positive’ (e.g., peer connection aspects) components of peer relationships can be associated with greater low mood and anxiety [51, 52] and that girls can engage in co-rumination in problematic ways [53]. Evidence on the relationship between social media and adolescent mental health remains mixed, yet participants’ accounts of aspects of social media engagement as promoting insecurity (and in turn affecting mental health) echoes theoretical and empirical literature indicating that such aspects may be especially problematic for girls [54]. Thus, our study complements the wider evidence base to corroborate understandings of risk factors for adolescent girls’ low mood and anxiety.

Our study adds insights and nuances that deepens knowledge and understanding regarding low mood and anxiety among adolescent girls, and can direct further research. This includes contributing evidence where youth insight on the link between a given factor and low mood and anxiety is lacking, such as academic attainment, where much qualitative evidence in mental health implications stems from adults supporting adolescent girls, rather than girls themselves [25]. This finding reiterates the need for researchers, policymakers, and practitioners to engage carefully with questions of how academic pressure is affecting young people [55], including girls in particular. Another insight relates to how structural issues are experienced by girls in England; for instance, frustrations with gendered norms, and the depth of implications for self-concept, are well-established in feminist literature [56], and our findings offers an important layer of further insight into the depth of how these norms are felt by the current generation of adolescent girls in England. This highlights the need for schools and other community and youth settings to focus attention on addressing systemic gender norms, such as gendered discourses in classrooms and the ways that sexual harassment are inherently permitted [57]. Notably, some of the explanations raised by our participants are not necessarily entirely specific to current cohorts. Reiteration of gender norms is an evolving rather than new challenge, and difficult aspects of peer relationships are common in adolescent friendship and indeed can contribute to psychosocial development [58]. Yet certainly some factors, such as academic pressure and social media, can evolve substantially over time, and all such factors can be relevant for understanding the persistent gender gap in low mood and anxiety *and* apparent increases over time.

As explored throughout various themes, and particularly in the final theme (Theme 6, *There is no easy answer to explain or resolve issues*), participants drew attention to the interwoven, complex nature of various issues, and cautioned against reductionism. This interwovenness was most clear for comparative aspects of social media, which appeared to act as an additional platform for other processes (e.g., gendered expectations and comparison to peers) to play out and be magnified. This echoes discussions around how social media functions intersect with wider developmental contexts of adolescence, fostering a focus on body image and appearance at a time of identity exploration and heightened investment in peer approval [54], and demonstrates a need for responses that match this complexity.

Spanning several of the explanations put forth were proposed underlying mechanisms of stress and pressure as well as self-appraisal. This is important, as it has been consistently suggested that worsened low mood and anxiety among girls are grounded in ‘chronic strain’, or a prolonged, persistent experience of daily stressors that, cumulatively, create a continuous state of tension leading to worsened mental health [1, 59]. For self-appraisal, studies have generally indicated that girls experience lower self-esteem than boys, especially in adolescence, although this is dependent upon conceptualisation, domain, and nuances such as the extent of internalisation of external norms [60]. Participants’ consistent identification of similar underlying mechanisms emphasises the need for further understanding of the mechanisms by which both individual and compounded risk factors are associated with heightened low mood and anxiety among girls.

This study holds critical policy and practice significance, offering insights that can be explored contextually and in conjunction with wider knowledge and understanding to extend and enhance systems and interventions to best meet girls’ needs. Structurally, for instance, our discussions with girls suggest a need to challenge the gendered discourses communicated to children and adolescents; review the educational pressures of UK secondary education; provide education and support relating to peer relationships; and evaluate social media functionality and engagement for young people. More directly, our analysis highlights a need for those working with adolescent girls to create supportive, open spaces for nuanced conversations about the challenges they face, together with opportunities to bolster self-esteem and coping capacities. In research, policy, and practice, efforts to progress the agenda to improve girls’ mental health can be best achieved through engagement with girls as key contributors to decision-making, as has been a key strength in our study; see, for instance, guidance and publications around conceptualising and reporting on co-produced health interventions and services [61, 62].

### Strengths and limitations

This study offers a conceptually and methodologically important contribution to knowledge and understanding on gendered inequalities in low mood and anxiety. A key strength is our emphasis upon youth voice, both through a qualitative design eliciting adolescent girls’ perceptions and through our co-production of the study with young women, both of which are rarely included in work in this area. Participants appeared enthused in sharing their views and found resonance in collectively unpacking these, leading to rich data with important implications for ongoing research and for public health efforts. Young researchers played a critical role in designing, guiding, and interpreting discussions, and had invaluable input in contextualising and making sense of the challenges that participants raised. The study can therefore impart youth voice into efforts to unpack gendered mental health inequalities and demonstrates the fruitfulness of engagement with adolescent girls within this area.

However, this is a perception-based study undertaken in group contexts where some points may have been dominant or unelicited, and was ‘light touch’ in that we met participants once and asked them not to share things that might feel upsetting or personal. Thus, we cannot necessarily clarify the specific nature of potential contributing factors (i.e., how these function statistically in epidemiological risk analysis) or specificity to adolescent girls (e.g., that the issues explored are not similarly experienced by boys), timing may have affected the focus and depth of discussion (e.g., focus groups were in exam season, perhaps bringing the role of educational pressures into sharper focus). This might explain why, for instance, COVID-19 as an influence was discussed but not necessarily framed as a major concern; we did not directly ask about this, and our framing of issues over their adolescent years may have encouraged them to cast their mind over a wider period of time, given that they were late adolescents in 2022. Though the purpose of the study was to understand girls’ perspectives on rates of low mood and anxiety among their population, necessitating the introduction of evidence into discussions, it is possible that more spontaneous or neutral discussion of girls’ mental health may have taken discussions in other directions. This study can be complemented by more immersive qualitative investigation such as one-to-one interviews or ethnographic exploration in schools and other community and youth spaces, and longitudinal research examining risk factors and mechanisms for low mood and anxiety over time. Though we saw engagement with girls from varying ethnic and religious backgrounds and from LGBTQIA+ communities, those who have been eligible for free school meals or who are disabled or transgender were less represented. Further exploration with adolescent girls from particular backgrounds and communities will be valuable, particularly given the intersectional nature of inequality. This includes engagement with girls of different ages – including those still in the earlier stages of adolescence – and with girls in different countries and cultural contexts to interrogate this as a global issue.

## Conclusion and implications

Identifying and understanding the potential contributors to low mood and anxiety as perceived by adolescent girls themselves – namely narrow expectations for girls and women, intense educational pressures, challenges in peer relationships, and comparison and insecurity in social media – is a critical contribution to literature given limited evidence of their views, particularly in a UK context and in recent years. Our study can direct future research in numerous ways, including providing youth-informed direction for epidemiological research examining risk factors, and more targeted research to illuminate the contexts, processes, and mechanisms wherein particular experiences are associated with low mood and anxiety for girls. We recognise principles of equifinality and multifinality and acknowledge that many adolescent girls face a range of wider psychosocial factors that contribute to their mental health (and indeed, better understanding of *protective* factors for girls would be valuable). Yet key issues raised here resonate with broader concerns within the field of youth mental health. For instance, workshops across 2019 and 2020 with academics, practitioners, policymakers, and young people identified research priorities for youth public mental health that included social media, school culture and the focus on attainment, and guidance for peers on how to best support one another [63]. Research, policy, and practice efforts in relation to adolescent mental health therefore have the potential to yield substantial benefits for a wide-ranging audience beyond only girls, but also should be approached with a nuanced understanding of gender-specific contexts and processes. This is particularly important to inform and mobilise a strategic response to high rates of low mood and anxiety among girls as a key public health issue.

## Electronic supplementary material

Below is the link to the electronic supplementary material.

Supplementary Material 1

## Data Availability

Data cannot be made available due to ethics and privacy restrictions in line with the nature of our consent with our participants. All data generation materials from the project are publicly available via the Open Science Framework: https://osf.io/kbta4. We have shared a list of all codes underpinning our themes in the Supplementary Materials of this article.

## Abbreviations

RTA: Reflexive thematic analysis
STEM: Science, technology, engineering, and mathematics
