# Supplementary material for: Adolescent girls’ explanations of high rates of low mood and anxiety in their population: a co-produced qualitative study
Source: BMC Womens Health. 2025 Feb 4;25:49. doi: 10.1186/s12905-024-03517-x (PMC11792720; doi:10.1186/s12905-024-03517-x)
Supplement: Supplementary file 1 — Supplementary Material 1 [file 12905_2024_3517_MOESM1_ESM.docx]

**Supplementary Materials**

**A. Overview and reflections on co-production**

| **Box A: Overview and reflections on co-production**  *Guided by the GRIPP-2 short-form reporting tool (Staniszewska et al., 2017)* |
| --- |
| **Aims:** to embed young people’s perspectives throughout project design, implementation, and interpretation, embedding youth voice to facilitate us to better engage with our participants and more meaningfully interpret their experiences. |
| **Methods: T**hroughout, we worked with Common Room North Ltd, a consultancy organisation focused on embedding youth voice into mental health policy, research, and practice. During initial development, we engaged in group discussion with seven young women aged 18–22 from diverse backgrounds. This discussion was advertised via Common Room North’s various networks and platforms and interested individuals were invited to get in touch. Once awarded funding, we recruited for young researchers, with Common Room North sharing an advert for the role along their networks; interested candidates applied and were interviewed. Our young researchers (PN and JL) then joined the core team and were active members in all stages, including: a) developing recruitment and data generation approaches to ensure these were understandable, engaging, and inclusive; b) co-leading focus groups alongside a university researcher with training and regular debriefing; c) analysing data, with one young researcher reviewing coding and co-developing themes, and the other reviewing themes; and d) planning and contributing to dissemination. We sought to balance young researchers’ input, involving them throughout the process but minimising undue burden; they undertook an average of two days a month of work across the course of the project. We held training workshops in relation to data generation (exploring good practice in qualitative processes and focus groups, reviewing the schedule together, holding a mock focus group), and data analysis (discussing the principles of qualitative analysis and reflexive thematic analysis, reviewing coding together, and guidance on using NVivo). |
| **Outcomes and extent of influence on the study:** Together we reflected and learned ‘on the go’ to continually strengthen our co-production approaches and wider procedures (e.g., reflecting on how focus groups were running). Our young researchers’ input in shaping materials and processes led to these being more engaging and inclusive, and we felt that their active role in focus groups lessened power dynamics and supported engagement. Our analytic discussions added further nuance to interpretations, partly as our young researchers were closer to more contemporary adolescence experiences. |
| **Reflections and critical perspectives:** The whole team embraced co-production, recognising its value and working to ensure accessibility (e.g., avoiding jargon). We worked collaboratively to navigate mutual respect and disagreement, ensuring young researchers felt able to speak up, and safe to have their suggestions critically explored. One potential pitfall we explored was ensuring young researchers did not become seen as spokespeople for all young people, particularly as in later stages of the project one young researcher was more heavily involved; one lesson may be to assemble a slightly larger team of young people, which might dissuade such default thinking. Another challenge was navigating university structures; as young researchers were employed via Common Room this limited such issues, but there were barriers, such as gaining certain IT system access. |

**B. Codes underpinning analysis**

| **Themes and nested codes** | **Transcripts**  **(8 total)** | **References** |
| --- | --- | --- |
| **A. Mental health context right now** | 8 | 146 |
| a. Mental health issues and reporting increasing | 8 | 83 |
| Agree that rates in girls are increasing | 1 | 1 |
| Agrees that rates are increasing | 1 | 1 |
| Agrees with increase | 1 | 1 |
| COVID feeding into current mental health (especially for girls) | 6 | 53 |
| Boys are more used to socialising communicating virtually | 1 | 1 |
| Busy lives distracts people from thoughts | 1 | 1 |
| Coming out of lockdown was overwhelming | 1 | 1 |
| Communicating via phone not as rewarding as face-to-face communication. | 1 | 2 |
| Communication over the phone is different to face-to-face communication | 1 | 1 |
| Confusing information about how assessments are happening | 1 | 1 |
| Covid as feeding into girls' difficulties | 1 | 1 |
| COVID confinement = unable to get support from teachers | 1 | 1 |
| COVID confinement in schools led to anxiety | 1 | 1 |
| Covid gave people chance to process events that happened | 1 | 1 |
| COVID handled well in private school | 1 | 1 |
| COVID has increased mental health issues among teenagers | 1 | 1 |
| Covid has less impact on the way boys communicated with each other | 1 | 1 |
| Covid impacted normal communication between girls | 1 | 1 |
| COVID impacted teenagers more | 1 | 1 |
| Covid impacting mental health | 1 | 1 |
| Covid led to revisiting events and the emotions that went with it | 1 | 1 |
| Covid seen as contributing to increasing rates | 1 | 1 |
| Drastic change in socialising made girls lonely | 1 | 1 |
| Games with friends but not same as socialising face-to-face | 1 | 1 |
| Girls had to adopt different ways of socialising than they usually would during lockdown | 1 | 1 |
| Girls prefer socialising face-to-face | 1 | 1 |
| Had to get extra support during covid | 1 | 1 |
| In lockdown for the most important time at school | 1 | 1 |
| Lack of physical affection during covid contributed to low mood | 1 | 1 |
| Lockdown and school closures have affected young people | 1 | 1 |
| Lockdown brought push to talk about feelings, ask for help | 1 | 1 |
| Lockdown gave people time to think | 1 | 1 |
| Lockdown knocked young people's confidence and socialising | 1 | 1 |
| Lockdown made girls lonely | 1 | 1 |
| Lockdown meant that people had more time to re-surface past traumas and experiences | 1 | 1 |
| Missing social time due to COVID | 1 | 1 |
| No choice in form of communication except over the phone during lockdown | 1 | 1 |
| Not being able to see friends and family caused low mood | 1 | 1 |
| Nothing to distract people from their thoughts | 1 | 1 |
| Online social communication is different to face to face communication | 1 | 1 |
| Poor experiences of COVID and lockdowns | 1 | 1 |
| Returning to school was a shock | 1 | 1 |
| Rumination led to diminished MH | 1 | 1 |
| School moved on too quickly from covid to exams | 1 | 1 |
| Schools could have handled COVID better | 1 | 1 |
| Schools handled COVID poorly | 1 | 1 |
| Socialising in person is important | 1 | 1 |
| Socialising in person is important for girls | 1 | 1 |
| Society hasn’t stopped to process impact of covid | 1 | 2 |
| Things have moved on from COVID too fast | 1 | 1 |
| Thinking about things that you could ignore in ‘normal’ life | 1 | 1 |
| Time alone led to rumination of event they hadn’t processed | 1 | 1 |
| Time alone led to rumination of event they hadn’t processed (2) | 1 | 1 |
| Unable to talk about things because of lockdown | 1 | 1 |
| Would benefit from talking about impact of covid at school | 1 | 1 |
| Higher rates aren't surprising at all | 1 | 2 |
| Increase doesn't surprise me | 1 | 1 |
| Increase echoes wider messaging around mental health problems increasing | 1 | 1 |
| Increase isn't surprising | 1 | 1 |
| Increase makes sense in line with social media | 1 | 1 |
| Increase not a surprise - wouldn't expect anything other | 1 | 1 |
| Increased rates reflect genuine increase AND openness | 1 | 1 |
| Increasing rates 'make a lot of sense' | 1 | 1 |
| It's normal seeing low mood and anxiety right now | 1 | 1 |
| Lots of friends experience low mood and anxiety | 1 | 1 |
| Mental health issues are common amongst peers | 1 | 1 |
| MH issues have increased but always been there | 1 | 1 |
| More factors contributing to poorer mental health now compared to in the past | 1 | 1 |
| More things impacting MH than in past | 1 | 1 |
| More to be worried about than there used to be | 1 | 1 |
| Not shocked by increase | 1 | 1 |
| Not suprised by increased rates | 1 | 1 |
| Not surprised by increase | 1 | 1 |
| Not surprised by the increase in rates | 1 | 1 |
| Not surprised to hear of increase | 1 | 1 |
| Poor mental health is rising | 1 | 1 |
| Reported increase reflects own perceptions | 1 | 1 |
| Rising rate of distress has become the norm | 1 | 1 |
| Sad to hear of increase | 1 | 1 |
| Seen increases in rates in friends from conversations | 1 | 1 |
| Would have expcted higher rates | 1 | 1 |
| b. The ways we talk about mental health | 6 | 63 |
| Everyone has mental health (difficulties) | 3 | 13 |
| Everyone has mental health but not everyone has serious difficulties | 1 | 1 |
| Everyone I know struggles with MH problems | 1 | 1 |
| Everyone is having to deal with their own mental health | 1 | 1 |
| Help only available for people with 'serious' MH issues | 1 | 1 |
| It's recognised that everyone has MH | 1 | 1 |
| Mental health problems are more common than we think | 1 | 1 |
| MH is as normal as going through puberty | 1 | 1 |
| Most people in their school suffer from something with mental health | 1 | 1 |
| Most people struggle with mental health | 1 | 2 |
| Normality of low mood & anxiety | 1 | 1 |
| Not speaking about all parts leads to people wrongly self-diagnosing | 1 | 1 |
| Perception that it is normal for teenage girls to have low mood | 1 | 1 |
| Gaps in understanding | 4 | 11 |
| Found it weird that others didn't understand panic attacks | 1 | 1 |
| Generation differences | 1 | 1 |
| Generational differences | 1 | 1 |
| Generational differences (2) | 1 | 1 |
| Generational divides necessitate support | 1 | 1 |
| Lack of awareness of variety of MH issues | 1 | 1 |
| Lack of understanding about MH issues | 1 | 1 |
| Lessons on mental health are not relatable | 1 | 1 |
| Parents may struggle to understand MH issues | 1 | 1 |
| Some MH health issues are well known; others not | 1 | 1 |
| There's lots of MH awareness but not much awareness of ways to help each other | 1 | 1 |
| Normalisation and openness (and inherent problems) | 4 | 25 |
| Dismissive - 'everyone is going through something' | 1 | 1 |
| MH has been romanticized | 1 | 2 |
| MH issues need to be normalised to reduce stigma | 1 | 2 |
| MH more normalised than in previous generations | 1 | 1 |
| MH spoken about more in school | 1 | 2 |
| More comfortable talking about MH | 1 | 2 |
| More people are willing to talk about mental health | 1 | 1 |
| Needs to be less sympathy towards MH | 1 | 1 |
| Normalising difficulties | 1 | 1 |
| Normalising these issues would make people happier | 1 | 1 |
| Nothing wrong with you unless you’re abused or suicidal | 1 | 1 |
| People are more open about mental health than they used to be | 1 | 1 |
| People want MH issues because they dont know about 'ugly' parts | 1 | 1 |
| People want to have MH issues | 1 | 2 |
| Perception that it is normal for teenage girls to have low mood | 1 | 1 |
| Sympathy towards MH issues makes it not normal | 1 | 1 |
| Talking about MH not a bad thing | 1 | 1 |
| You’re not important unless something terrible is happening to you | 1 | 1 |
| YP make jokes about their MH to each other (coping) | 1 | 2 |
| Stigmas and insensitivity | 2 | 14 |
| 'okay not to be okay, until it gets messy' | 1 | 1 |
| 'ugly' parts of MH aren't spoken about | 1 | 2 |
| Avoid seeking help due to low self-esteem and stigma | 1 | 1 |
| Can't tell people about the 'ugly' parts of MH | 1 | 1 |
| Issues are openly spoken about, but still get judged admitting you have it | 1 | 1 |
| Mental health issues positioned as weakness in school lessons | 1 | 1 |
| Mental health sessions insensitive - change your mindset! | 1 | 1 |
| Mental health support feels robotic and this is offputting | 1 | 2 |
| Mental health support that already exists lacks compassion and individualisation | 1 | 1 |
| More awareness and less stigma needed for lesser known MH issues | 1 | 1 |
| Need to talk about every part of MH | 1 | 1 |
| Needs to be less shame for 'ugly' parts of MH | 1 | 1 |
| **B. How girls and women 'should' look and behave** | 8 | 382 |
| a. Sexist stereotypes and expectations | 8 | 77 |
| A general sense of gendered expectations and stereotypes | 8 | 18 |
| Competition & pressure comes from ingrained prejudice towards women | 1 | 1 |
| Expectations of what women should be good at | 1 | 1 |
| Expectations to uphold | 1 | 1 |
| Gendered expectations around behaviour | 1 | 1 |
| Help address gender stereotypes | 1 | 1 |
| Judgement of women | 1 | 1 |
| Labelling things as femininemasculine is a problem | 1 | 1 |
| Mindsets on how girls and boys should behave need to be addressed through education | 1 | 1 |
| Mysogyny dictating how women should behave | 1 | 1 |
| Negative mindset toward women | 1 | 1 |
| No personal experience of this - expectations are already there | 1 | 1 |
| Pressure on girls is immersed in our society | 1 | 1 |
| Provide the idea; encourages changes in attitudes | 1 | 1 |
| Raising awareness of gendered issues for women | 1 | 1 |
| Reduce bias towards women | 1 | 1 |
| School inequalities reflect wider social inequalities | 1 | 1 |
| Society and pressure | 1 | 1 |
| Spoken and unspoken expectations of how women should act | 1 | 1 |
| Beauty and appearance expectations | 6 | 21 |
| Beauty standards on women | 1 | 1 |
| Because men are 'superior' this creates pressure for women to step up via appearance | 1 | 1 |
| Emphasis on women having a youthful appearance | 1 | 1 |
| Expectations in society on how women should look | 1 | 1 |
| Expectations on what girls should wear | 1 | 1 |
| Gender stereotypes impact expectations on clothing | 1 | 1 |
| Gendered expectations around diet and physicality | 1 | 1 |
| Girls expected to look ‘feminine’ | 1 | 1 |
| Homogeneous beauty expectations - everyone should look a certain way | 3 | 10 |
| Everyone can’t look the same | 1 | 1 |
| Expectation to look a certain way comes from how men treat women | 1 | 1 |
| Expectations of 'perfection' | 1 | 1 |
| Forced to play into beauty standards | 1 | 1 |
| Girls are expected to dress in a certain way | 1 | 1 |
| Have to look one way or else you are ugly | 1 | 1 |
| Not being able to find your own way of presenting yourself | 1 | 1 |
| Not getting to choose what is beautiful to you | 1 | 1 |
| Online images lead girls to think they have to live and look a certain way | 1 | 1 |
| Pressure on girls to focus on appearance and look a certain way | 1 | 1 |
| Not allowed to be natural | 1 | 1 |
| Pressure to actively change how you look | 1 | 1 |
| Subtle changes in expectations on how women should look all the time | 1 | 1 |
| Complexities | 3 | 3 |
| Different ways of being a girl - and none of them free of judgement | 1 | 1 |
| Discrepancy between the pressure to do well and societies limits on girls | 1 | 1 |
| Gender stereotypes are more restrictive for women | 1 | 1 |
| Difficult to challenge | 3 | 5 |
| 'Laddish' culture might stand in the way of progress | 1 | 1 |
| Difficulty changed oldfashioned mindset | 1 | 1 |
| It is difficult to move past judgements of women | 1 | 1 |
| It is difficult to move past judgements of women (2) | 1 | 1 |
| People may not see their own bias toward girls | 1 | 1 |
| Expectations that girls be quiet and polite | 4 | 7 |
| 'Girls should be quiet' | 1 | 1 |
| Boys are able to be in charge and aggressive - girls have to be quiet | 1 | 1 |
| Boys are encouraged to be confident - girls told to be quiet, reserved | 1 | 1 |
| Boys grow up seeing women as 'quiet' | 1 | 1 |
| Girls should be polite, feminine and shouldn't talk about issues | 1 | 1 |
| Pressure to act in a way that pleases others over themselves | 1 | 2 |
| Stereotypes and mindsets as longstanding and ingrained | 5 | 12 |
| Gender views have improved but still prevalent | 1 | 1 |
| Mindset from stereotypes lasts until taught differently | 1 | 1 |
| Mindset r.e. stereotypes changes but expectation on women don't | 1 | 1 |
| Need to educate everyone not just children | 1 | 1 |
| Old fashioned stereotypes of women still impact | 1 | 1 |
| Older generations 'stuck in their ways' | 1 | 1 |
| Older generations need to be aware of change | 1 | 1 |
| Perceptions of women are grounded in longheld patriarchal values and beliefs | 1 | 1 |
| Society needs to unlearn gender biases | 1 | 1 |
| Steroetypes leads to ingrained mindset of what you can be as a woman | 1 | 1 |
| Success = reducing gender stereotypes | 1 | 1 |
| Traditional, old fashioned views of girls and women's behaviour has prevailed | 1 | 1 |
| View of men as superior (and therefore favoured and better respected) | 4 | 11 |
| Because men are 'superior' this creates pressure for women to step up via appearance | 1 | 1 |
| Boys and men are favoured over girls | 1 | 1 |
| Girls seen as lesser and less intelligent | 1 | 1 |
| Less respect towards women than men | 1 | 2 |
| Men are seen as superior | 1 | 1 |
| Normalised view of women as inferior | 1 | 1 |
| Prevailing concept of men as better | 1 | 1 |
| Showing girls that they are just as valued (and their growth and education) | 1 | 1 |
| Traditional views of men as more capable | 1 | 1 |
| Unconscious view that men know better than women | 1 | 1 |
| b. Consistent communication and reinforcing of expectations | 8 | 163 |
| Boys encouraged in ways that girls are not | 3 | 12 |
| Boys are encouraged and girls are limited | 1 | 1 |
| Boys are encouraged to be confident - girls told to be quiet, reserved | 1 | 1 |
| Boys are raised to be leaders, and girls are not | 1 | 1 |
| Boys given options of what they can be | 1 | 1 |
| Boys' sports favoured over girls' | 1 | 1 |
| Emphasis on boys' sports, not girls sports | 1 | 1 |
| Encouraging girls' voices in the classroom | 1 | 2 |
| Girls less encouraged into sports | 1 | 1 |
| Girls' sports don't matter | 1 | 1 |
| Normalise sports engagement for girls (including in advertising and engaging) | 1 | 1 |
| Not necessarily that teachers favour boys (though some do) | 1 | 1 |
| Boys take on and reiterate expectations | 3 | 4 |
| Boys build high expectations of girls | 1 | 1 |
| Boys have narrow views on how girls should behave | 1 | 1 |
| Boys take on body image expectations of girls and put them on girls | 1 | 1 |
| Girls may project insecurities onto other girls based on what boys pervceive as attractive | 1 | 1 |
| Gendered expectations in learning spaces | 3 | 5 |
| Challenging teacher bias (e.g., in STEM, or leadership behaviours) | 1 | 1 |
| More different types of people in girl schools | 1 | 1 |
| Need for acknowledgement of gender bias in educational settings | 1 | 1 |
| People are more accepting to difference in girls schools | 1 | 1 |
| Stereotypes impact on every aspect of school life | 1 | 1 |
| How people respond to you | 7 | 42 |
| ALL ways of being are judged for girls | 3 | 5 |
| Girls are made fun of whatever they like | 1 | 1 |
| Girls are representatives of girls - boys are just themselves | 1 | 1 |
| Girls can't win - they're judged whatever they do | 1 | 1 |
| Girls constantly worried about being judged | 1 | 1 |
| Girls should be able to act how they want - but they are judged for it | 1 | 1 |
| Feeling stereotypically generalised as girls | 1 | 1 |
| Have to act more masculine to be taken seriously in a male dominated field | 1 | 1 |
| Judged for not conforming in some way | 7 | 29 |
| Adults commented on less feminine clothing at young age | 1 | 1 |
| Adults further engraining gendered stereotypes | 1 | 1 |
| Behaviour interpreted as 'bossy' | 1 | 1 |
| Being made fun of for doing 'boy' things | 1 | 1 |
| Boys get uncomfortable when girls don't conform to gender norms | 1 | 1 |
| Can’t be friends with girls because I’m seen as manly | 1 | 1 |
| Deviating from normalised girl expectations can become challenging | 1 | 1 |
| Does both typically male and female sports | 1 | 1 |
| Girls are called bossy in group contexts | 1 | 2 |
| Girls judged when wearing trousers | 1 | 1 |
| Girls liking football attributed to crush on boys | 1 | 1 |
| Girls made fun of for 'non-female' hobbies | 1 | 1 |
| Girls seen as bossy or complaining when talking about MH issues | 1 | 1 |
| Girls' behaviour is interpreted differently to when a boy does it - like leadership | 1 | 1 |
| Has masculine hobbies | 1 | 1 |
| History and patriarchy continue to affect behaviour and judgements | 1 | 1 |
| Interpretations of girls liking 'boy things' | 1 | 1 |
| Judgement on how girls should act | 1 | 1 |
| Less gendered judgement in girls schools | 1 | 2 |
| Liking football = tomboy | 1 | 1 |
| May not realise you are acting more masculine | 1 | 1 |
| Not being interested in boyfriends brings pressure and anxiety | 1 | 1 |
| People treat you differently depending on how you express your gender | 1 | 1 |
| Seen as manly by boys because of ‘boyish’ sports | 1 | 1 |
| Speaking out as a female leads to stereotyping | 1 | 1 |
| Women are looked down on for doing a ''man's'' job | 1 | 1 |
| You stand out if you don't wear what is expected | 1 | 1 |
| Not taken seriously if you don’t become more masculine | 1 | 1 |
| People find it easier to take frustrations out on women | 1 | 1 |
| Teachers commenting on girls' appearance - 'is it to impress a boy' | 1 | 1 |
| Women are slandered and judged more than men | 1 | 1 |
| Women have to be more masculine to progress | 1 | 1 |
| You should smile, and if you're not you get told to smile | 1 | 1 |
| Media portrayals of women | 6 | 20 |
| Archaic representations of girls and women in media and society | 1 | 1 |
| Femininity portrayed as negative | 1 | 1 |
| Hard to relate to women in the media | 1 | 1 |
| Internationalisation of body image 'norms' and standards | 1 | 1 |
| Lack of non-feminine women role models in the media | 1 | 1 |
| Media as a key contributor | 1 | 2 |
| Media as a negative presence in portraying women | 1 | 1 |
| Media creates insecurity | 1 | 1 |
| Media has a large focus on women's appearance | 1 | 1 |
| Media has a large focus on women's personality and comparisons to men | 1 | 1 |
| Media only presents one way of looking at gender | 1 | 1 |
| Media portrays gendered behaviours for girls | 1 | 1 |
| Media presents women in same way | 1 | 1 |
| Media putting expectations on how women should be and what they should do | 1 | 1 |
| Movie culture impacts how girls act | 1 | 1 |
| Movie culture sets ingrained stereotypes | 1 | 1 |
| Poor portrayal of women and femininity in media (movies, songs, etc) | 1 | 1 |
| Problematic media portrals of women | 1 | 1 |
| The media advertises a 'certain standard' | 1 | 1 |
| Messaging and pressure about appearance | 4 | 9 |
| Appearance is shoved towards girls | 1 | 1 |
| Because men are 'superior' this creates pressure for women to step up via appearance | 1 | 1 |
| Constantly changing beauty expectations cause worry | 1 | 1 |
| Diets encouraged at age 13 to girls specifically | 1 | 1 |
| Eating at school is a contributor | 1 | 2 |
| Eating disorders 'fashionable' at school | 1 | 1 |
| Exposed to unachievable beauty standards | 1 | 1 |
| Girls taught appearance is important | 1 | 1 |
| Messaging embedded from early age | 6 | 15 |
| Boys and girls together in primary schoo; | 1 | 1 |
| Early segregation = gender stereotypes | 1 | 1 |
| Gender expectations are communicated to young children | 1 | 1 |
| Gendered messaging growing up | 1 | 1 |
| Girls and boys are raised differently and that has long-term implications | 1 | 1 |
| Girls are taught how to behave in a feminine way from a young age | 1 | 1 |
| Girls immediately put into gender typical role | 1 | 1 |
| Having clubs is needed when gender issues are already in place - if this could be dealt with earlier it mightn't be needed | 1 | 2 |
| Stereotypes being put on girls at young age | 1 | 1 |
| Stereotypical gendered toys lead to focus on appearance | 1 | 1 |
| Stop early segregation between genders | 1 | 2 |
| Taught looks matter from young age | 1 | 1 |
| Young boys portray a type of 'attractive' women | 1 | 1 |
| Narratives that counter this | 3 | 8 |
| Encourage women to be more open to showing their true selves | 1 | 1 |
| Following body positivity accounts | 1 | 1 |
| Helping girls understand they don't need to conform to standards | 1 | 1 |
| Makes women more comfortable and heard | 1 | 1 |
| Need to see body positivity among peers | 1 | 1 |
| Positive role models to tackle unrealistic expectations | 1 | 1 |
| Taking the focus away from appearance (face and body - not outfits) | 1 | 1 |
| There is more awareness and challenge of gendered expectations for girls and women | 1 | 1 |
| Pervasive messaging in daily life | 4 | 5 |
| Expectations for girls are more 'undercover' - more subtle than for boys | 1 | 1 |
| Gendered expectations and pressured as part of everyay life | 1 | 1 |
| Gendered expectations are ingrained without you knowing | 1 | 1 |
| Norms of how to act and look are pervasive in daily life | 1 | 1 |
| Seeing an image repeatedly leads to unconscious internalisation of images (2) | 1 | 1 |
| Pressure | 4 | 5 |
| Pressure and expectations about appearance | 1 | 1 |
| Pressure on girls to act and look certain way | 1 | 1 |
| Pressure on girls to focus on appearance and look a certain way | 1 | 1 |
| Pressure to 'perform' like you are something you are not | 1 | 1 |
| Pressure to have a boyfriend from quite young | 1 | 1 |
| Seeing how women are represented (and not) in society | 6 | 18 |
| Abortions being illegalized in America | 1 | 1 |
| America banning abortion highlights women not heard | 1 | 1 |
| Feminism has negative connotations | 1 | 1 |
| Government sympathise with women but no action to help | 1 | 1 |
| Growing up into a world that isn't accepting and welcoming (of women) | 1 | 1 |
| Inequality in medical knowledge about women's health | 1 | 1 |
| Lack of representation for women in Government | 1 | 1 |
| Optimism that boys will speak out for girls | 1 | 2 |
| Outer society issues add stress | 1 | 1 |
| Society has job type expectations for men and women | 1 | 1 |
| Success = boys speaking out against inequality | 1 | 2 |
| Success = men showing support for women | 1 | 1 |
| Teenage girls are becoming more aware of the world (and things affecting women) | 1 | 1 |
| Want men to speak out against injustices towards women | 1 | 1 |
| Women aren't heard in Government | 1 | 1 |
| Women's rights are being attacked | 1 | 1 |
| Social media and celebrity culture reinforce beauty expectations | 5 | 20 |
| A lot of influencers getting cosmetic surgeries | 1 | 1 |
| Beauty standards are encouraged by celebrity culture | 1 | 1 |
| Celebrity culture increasing pressure to be better than everyone | 1 | 1 |
| Editing on social media impacts body image | 1 | 2 |
| Inappropriateness of comparing self to grown women at 16 | 1 | 1 |
| Online images lead girls to think they have to live and look a certain way | 1 | 1 |
| Peer pressure around body image, fed by social media | 1 | 1 |
| People would feel more comfortable with themselves on social media if less people photoshopped their photos | 1 | 1 |
| Photoshopping is happening in the first place because of insecurity | 1 | 1 |
| Repeated imagery setting beauty standards | 1 | 1 |
| Social media impacting girls more because it’s more accessible | 1 | 1 |
| Social media sets body norms | 1 | 1 |
| Standards advertised by social media contribute to insecurities | 1 | 1 |
| What happens when your body is not standard in line with social media meessaging | 1 | 1 |
| Younger girls are heavily influenced by beauty standards portrayed in social media | 1 | 1 |
| younger girls are less likely to challenge or think about what they see on social media | 1 | 3 |
| Younger girls may not fully understand that social media is fake | 1 | 1 |
| c. Profound impact | 8 | 142 |
| Appearance judgements and insecurity | 8 | 52 |
| (some) Females want to be scouted by reality TV because of behaviour encouraged | 1 | 1 |
| Appearance determines worth | 1 | 1 |
| Appearance expectations are normalised - makeup, shaving | 1 | 1 |
| Being smart is compensation for not feeling you meet appearance expectations | 1 | 1 |
| Being smart vs. being beautiful | 1 | 1 |
| Body image is a major contributing factor | 1 | 1 |
| Boys judging bodies creates insecurity | 1 | 1 |
| Breakups impact body image | 1 | 1 |
| Bullying and image put on her made her feel bad about herself | 1 | 1 |
| Comparing to others leading to low self-esteem leading to low mood | 1 | 1 |
| Competition of who could eat the least (school) | 1 | 1 |
| Constant comparison to other; can't see good in self | 1 | 1 |
| Constantly changing beauty expectations cause worry | 1 | 1 |
| Cover up your flaws | 1 | 1 |
| Cycles of insecurity | 1 | 1 |
| Editing on social media impacts body image | 1 | 2 |
| Expectations leads to craving validation on looks | 1 | 1 |
| False standards become ingrained in you (whether you know it's fake or not) | 1 | 1 |
| Fear of judgement comes from focus on appearance | 1 | 1 |
| Feeling pressured to look a certain way | 1 | 1 |
| Feeling you are not similar to the 'attractive woman' that boys hold up as ideal | 1 | 1 |
| Financial pressure for those not able to match friends | 1 | 1 |
| Forced to play into beauty standards | 1 | 1 |
| Friends internalise body norms and then encourage them with each other | 1 | 1 |
| Girls are more focused on their looks in adolescence compared to boys | 1 | 1 |
| Greater impact of beauty standards on younger girls | 1 | 1 |
| Hard to match beauty standards | 1 | 1 |
| If you don't fit the beauty standard you feel worthless | 1 | 1 |
| It can feel belittling to not feel you match generic attractive ideals | 1 | 1 |
| Not being able to find your own way of presenting yourself | 1 | 1 |
| Not getting to choose what is beautiful to you | 1 | 1 |
| People have to conform – can’t be authentic self | 1 | 1 |
| Photoshopping is happening in the first place because of insecurity | 1 | 1 |
| Pressure to conform can be challenging if you feel different - cause overthinking, stress | 1 | 1 |
| Pressure to look and act certain way for girls causes anxiety in daily life | 1 | 1 |
| School culture of disordered eating led to hospitalisations | 1 | 1 |
| Secondary result of people getting surgery to meet beauty standard | 1 | 1 |
| Should I look like myself, or someone else | 1 | 1 |
| Social media impacting girls more because it’s more accessible | 1 | 1 |
| Starts with insecurities and gets bigger | 1 | 1 |
| Stereotypical gendered toys lead to focus on appearance | 1 | 1 |
| Those without money to make themselves look 'perfect' may feel especially bad | 1 | 1 |
| Wanting to fit in makes you want to change your appearance | 1 | 1 |
| Weight and appearance big factors in low self-esteem | 1 | 1 |
| What happens when your body is not standard in line with social media meessaging | 1 | 1 |
| Will be bullied by peers if girls don’t meet expectations | 1 | 1 |
| You are expected to shave and wear makeup | 1 | 1 |
| You don't get to actually make choices about how you want to look | 1 | 1 |
| You have to spend money to make yourself look perfect | 1 | 1 |
| You're not allowed to be natural | 1 | 1 |
| Younger girls are heavily influenced by beauty standards portrayed in social media | 1 | 1 |
| Deep impact on identity, self-concept, confidence | 7 | 23 |
| Boys are more confident than girls | 1 | 1 |
| Boys are more confident throughout life - because they're raised to be | 1 | 1 |
| Conforming leads to low mood – can’t be authentic self | 1 | 1 |
| Expectations on women lead to women masking who they are | 1 | 1 |
| Gender and confidence | 1 | 1 |
| Gender expectations affect confidence and feed into education pressure | 1 | 1 |
| Gendered behaviour towards girls affects confidence and self-esteem | 1 | 1 |
| Gendered expectations affect girls' confidence | 1 | 1 |
| Gendered expectations and inferiority happening during a formative time is a problem | 1 | 1 |
| Gendered expectations have long term implications | 1 | 1 |
| Girls and insecurity is treated as a joke, stereotype - but it's deeper than that | 1 | 1 |
| Girls carry childhood gender expectations with them through their lives | 1 | 1 |
| Girls filter and second guess themselves | 1 | 1 |
| Narratives of being lesser or not conforming are saddening | 2 | 2 |
| Being treated differently because of gender expression affects how you feel | 1 | 1 |
| Being viewed as inferior gets girls and women down | 1 | 1 |
| Normalised view of women as inferior makes girls and women feel worthless | 1 | 1 |
| Not being allowed to express yourself because of gender is normalised | 1 | 1 |
| other's opinions and views on how a girl should be hugely impacts girls' freedom to be who they want to | 1 | 1 |
| Should I look like myself, or someone else | 1 | 1 |
| Stereotypes make it difficult to find your own identity and relationship values | 1 | 1 |
| The way girls are viewed is dehumanising | 1 | 1 |
| Trying to be someone that's not you | 1 | 1 |
| Women can’t be themselves – can’t be authentic self | 1 | 1 |
| Narrowing of options for women | 5 | 13 |
| Compares control from enforced steroetypes to Hitler | 1 | 1 |
| Create same opportunities for men and women | 1 | 1 |
| Gender stereotypes limits women from a young age | 1 | 1 |
| Gendered career routes | 1 | 1 |
| Gendered expectations make girls less likely to try new things and take risks | 1 | 1 |
| Girls are shut out of activities that give them pleasure outside of social media | 1 | 1 |
| Girls should have same opportunities as boys | 1 | 1 |
| Less aware of 'male' subjects until college | 1 | 1 |
| Limits set on girls and women | 1 | 1 |
| Male dominated industries intimidating for girls | 1 | 1 |
| Never play football in all girl schools | 1 | 1 |
| Stereotypes limit girls | 1 | 1 |
| Steroetypes leads to ingrained mindset of what you can be as a woman | 1 | 1 |
| Normalisation, Unconscious internalisation through repetition | 2 | 3 |
| Appearance expectations are normalised - makeup, shaving | 1 | 1 |
| False standards become ingrained in you (whether you know it's fake or not) | 1 | 1 |
| Seeing an image repeatedly leads to unconscious internalisation of images | 1 | 1 |
| Pressures around having to fight and counter these narratives | 4 | 4 |
| Empowerment creating added pressure for girls | 1 | 1 |
| Feeling personal pressure on behalf of other women | 1 | 1 |
| More awareness of the wrongness of gendered expectations doesn't stop the pressure | 1 | 1 |
| Try to be the best at everything else to defend against judgements | 1 | 1 |
| Sexual harrassment | 6 | 47 |
| Barrier - if we start measuring sexual harrassment the numbers will be high even with this training because it's not recorded | 1 | 1 |
| Boys are confident to just harrass girls | 1 | 1 |
| Boys don't see you as a person | 1 | 1 |
| Boys in school affect girls' experiences and comfort | 1 | 1 |
| Boys ranking girls on their bodies | 1 | 1 |
| Boys' treatment of girls and consent is a source of stress | 1 | 1 |
| Cynical about educating boys on sexual harrassment - believe they won't listen | 1 | 3 |
| Discomfort with men commenting on looks and behaviour | 1 | 1 |
| Early education on sexual harrassment | 1 | 1 |
| Educate boys around sexual harrassment and gender issues | 1 | 1 |
| Educate teachers on how to handle sexual harrassment | 1 | 2 |
| Educate teachers on sexual harrassment | 1 | 1 |
| Harsher punishments for boys (with sexual harrassment) | 1 | 1 |
| Hetero relationships as vulnerable - boys share confidential things | 1 | 1 |
| Insensitive coverage of consent in school lessons | 1 | 1 |
| Issues of male gaze | 1 | 1 |
| Judging yourself as to whether boys find you attractive | 1 | 2 |
| Lessons on consent as victim blaming | 1 | 1 |
| Male gaze and women needing to be 'attractive' | 1 | 1 |
| Measuring success - If sexual harrassment behaviour is addressed it will stop | 1 | 1 |
| Men care more about women's appearance than women do about men | 1 | 1 |
| Men feel entitled to comment on apeprance and behaviour | 1 | 1 |
| Mistrust of boys in romantic relationships | 1 | 1 |
| More disciplining of boys for poor behaviour toward girls | 1 | 1 |
| Need for conversations about how boys should treat girls | 1 | 1 |
| Perception of sexual harrassment as flirting | 1 | 2 |
| Pressure to appear interested in a boyfriend | 1 | 1 |
| Punish and 'scare' boys who sexually harrass | 1 | 1 |
| Sessions on consent not helpful | 1 | 1 |
| Sexual harrassment as a common issue | 1 | 1 |
| Sexual harrassment in schools internalises internal dehumanisation | 1 | 1 |
| Sexual harrassment is embarrassing | 1 | 1 |
| Stricter punishment for sexual harassment in school | 1 | 1 |
| Support girls to protect themselves and build strong relationships | 1 | 1 |
| Survey young people on how their school ahndles sexism | 1 | 1 |
| Teachers blaming girls for sexual harrassment | 1 | 1 |
| Teachers should create consequences of sexual harrassment | 1 | 1 |
| Teased by boys for having masculine hobbies | 1 | 1 |
| Teasing from boys when taking part in traditionally masculine activities | 1 | 1 |
| Telling girls to prevent their own rapes | 1 | 1 |
| The best choice is to be independent (and not relying on a man) | 1 | 1 |
| Validation comes from being taught 'need men to survive' | 1 | 1 |
| **C. Intense educational pressures** | 8 | 263 |
| a. Multiple layers of educational pressure | 7 | 95 |
| COVID deepening educational pressures | 3 | 14 |
| 'we had to teach ourselves' | 1 | 1 |
| COVID and feeling less prepared for exams | 1 | 1 |
| COVID led to longer school days & higher workload | 1 | 1 |
| Dealt with lots of exams during COVID | 1 | 1 |
| Felt abandoned by teachers during lockdown | 1 | 1 |
| Had good grades until lockdown | 1 | 1 |
| Hard to catch up on things they have missed from COVID | 1 | 2 |
| More support for learning during covid needed | 1 | 1 |
| Not enough learning support during lockdown | 1 | 4 |
| Online lessons were too long & unengaging | 1 | 1 |
| Early high school years are very strict | 1 | 1 |
| Education as an alternative to achievement on social media | 1 | 1 |
| Exams and grades | 7 | 15 |
| Anxiety about exams | 1 | 1 |
| Anxiety caused by exams | 1 | 1 |
| Education and grades are a major focus for young people | 1 | 1 |
| Exam and career aspects are stressful | 1 | 1 |
| Exam stress can cause a lot of anxiety and creates pressure | 1 | 1 |
| Exam stress leading to low mood and anxiety | 1 | 1 |
| Exams are stressful and source of anxiety | 1 | 1 |
| Exams as a source of stress and affecting mental health | 1 | 1 |
| Pressure and exams | 1 | 1 |
| Pressure to get good grades | 1 | 1 |
| School and exam stress causes low mood and anxiety | 1 | 1 |
| unprepared for exams | 1 | 2 |
| Worry about grades puts you down | 1 | 1 |
| Worry about not getting into university | 1 | 1 |
| Expectations and being good enough | 6 | 24 |
| Academic pressure can lead to people feeling anxious | 1 | 2 |
| Atmosphere of competition in school | 1 | 1 |
| Don’t know if you’re good enough to get into university | 1 | 1 |
| Expectations and pressure to 'do well' | 1 | 1 |
| Feeling you're not living up to grade expectations | 1 | 1 |
| High achieving schools espcially bad for competitive atmosphere | 1 | 1 |
| Messaging that nothing is good enough in education | 1 | 2 |
| Not doing enough compared to peers | 1 | 1 |
| Not meeting grade expectation is demotivating | 1 | 1 |
| Pressure around standards and education - got to keep up | 1 | 1 |
| Pressure to get the best grades | 1 | 1 |
| Pressure to get the best grades (2) | 1 | 1 |
| School adds unrealistic and unnecessary expectations and pressure | 1 | 4 |
| School pressures are unrealistic | 1 | 1 |
| So much pressure (from peers) to get top grades | 1 | 1 |
| Some cultural input into high pressure around education | 1 | 1 |
| Those with parents who are immigrants experience more academic pressure | 1 | 1 |
| Wanting to meet expectations | 1 | 1 |
| Wants to be the best in what she does (pressure) | 1 | 1 |
| General concept of school as pressurising, stressful | 6 | 15 |
| Adapting to new environments - primary-secondary transition | 1 | 1 |
| Anxiety over homework workload | 1 | 1 |
| Breaking up long lessons to reduce intensity | 1 | 1 |
| College (and education) is really stressful | 1 | 1 |
| Education and school as a pressure for girls | 1 | 2 |
| High schools are the root of all evil | 1 | 1 |
| Intense academic pressure | 1 | 1 |
| Multiple things to worry about in college | 1 | 1 |
| School as a source of intense pressure in concert with wider life pressure | 1 | 1 |
| School as a source of pressure | 1 | 1 |
| School has burden and pressure | 1 | 1 |
| School is the biggest contributor low MH | 1 | 1 |
| The pressures and burden of school contributes to stress and low mood | 1 | 1 |
| Things at school are complicated to process | 1 | 1 |
| Lack of choice doesn't help | 1 | 8 |
| Forced to do subjects and activties they don't want to | 1 | 1 |
| Freedom to choose subjects they are interested in will help | 1 | 2 |
| Hated school when doing activities they disliked | 1 | 1 |
| Have to learn a language for GCSE | 1 | 1 |
| Process of picking subjects assumes everyone wants to do the same subjects | 1 | 1 |
| Recognition that it could be difficult to allow younger children to pick subjects | 1 | 1 |
| unfair if subjects clash | 1 | 1 |
| Making decisions | 4 | 9 |
| Huge pressure on young people to make life decisions | 1 | 1 |
| Lots of factors involved in deciding on further education | 1 | 1 |
| Picking options for college is hard | 1 | 1 |
| Pressure of what you'll do AFTER school | 1 | 1 |
| Schools need to remove some of the pressure - provide more information about the available options | 1 | 1 |
| Thinking about life plans and careers is stressful - has lots of expectations | 1 | 1 |
| Wanting to be aware of options other than university | 1 | 3 |
| Teacher narratives | 4 | 8 |
| Anxiety over getting reprimanded for not doing homework | 1 | 1 |
| Extreme messaging about exams and careers | 1 | 1 |
| Feeling teachers are presenting misinformation about educational options available - too narrow | 1 | 1 |
| Stop fear messaging around exams | 1 | 1 |
| Teachers assumed bad grades = lazy student | 1 | 1 |
| Teachers calling students out for missing homework | 1 | 1 |
| Teachers calling them out on grades was embarrassing | 1 | 1 |
| Teachers put a lot of pressure on young people | 1 | 1 |
| b. Pressure more acute for girls | 7 | 65 |
| Challenges in male dominated subjects | 5 | 33 |
| A need to prove to yourself you deserve to be in STEM studies | 1 | 1 |
| Boys are confident in STEM | 1 | 1 |
| Can’t be authentic self in male dominated fields | 1 | 1 |
| Doesn't take 'male' dominated subjects so doesn't feel pressure | 1 | 1 |
| Dynamic in relationships between teacher and boys in female subjects not as extreme | 1 | 1 |
| Environment in 'male' subjects impacting number of girls wanting to do subject | 1 | 1 |
| Environment in 'male' subjects not the same for girls | 1 | 1 |
| Extra pressure in STEM subjects due to men dominating | 1 | 1 |
| Feeling like you have to be the best in STEM | 1 | 1 |
| Feeling like you need to prove people wrong on gendered expectations (STEM) | 1 | 1 |
| Feminine girls not taken seriously in male dominated fields | 1 | 1 |
| Girls feel extra pressure in STEM studies | 1 | 1 |
| Girls limited to the type and depth of subjects | 1 | 1 |
| Girls may struggle more in more male-dominated subjects e.g. STEM | 1 | 1 |
| Girls put off from male dominated fields | 1 | 1 |
| Hard for women to progress in male dominated field | 1 | 1 |
| Harder for girls to get into male dominated subjects | 1 | 1 |
| In STEM there is lots of competition AND gendered pressure | 1 | 1 |
| Less girls in STEM (once no longer compulsory) | 1 | 1 |
| More pressure in classes with few girls | 1 | 1 |
| Needs to be more awareness towards women in medical field | 1 | 1 |
| Overwhelming for girls in 'male' subjects | 1 | 1 |
| Pressure not to conform to gendered stereotypes surrounding women in STEM | 1 | 1 |
| Pressure of male dominated subjects not as strong in all girls schools | 1 | 1 |
| Prevent areas being male dominated | 1 | 1 |
| Segregation undermines education on females going into male fields | 1 | 1 |
| Some subjects are male dominated | 1 | 1 |
| Studies or careers that are associated with men mean you have to be best of best if you pursue | 1 | 1 |
| Success = equality in careers | 1 | 1 |
| Teachers need to provide extra support to girls in male-dominated subjects | 1 | 1 |
| Very few girls in 'male' subjects at college | 1 | 1 |
| Women need to be the best of the best to be successful in male-dominated careers | 1 | 1 |
| Women need to try harder to get into male dominated fields | 1 | 1 |
| Gendered pressure, judgements, and narratives | 6 | 18 |
| Boys and girls both affected by school, but girls perhaps more | 1 | 1 |
| Boys are judged less for academic ability | 1 | 1 |
| Boys outshine girls in lessons - they're more confident | 1 | 1 |
| External pressure on girls in particular in education | 1 | 1 |
| Gendered views of women fuel a need to do the best in school | 1 | 1 |
| Girls experience more academic focus and judgement | 1 | 1 |
| Girls have to prove themselves | 1 | 1 |
| Girls pressured to be best in class | 1 | 1 |
| More pressure on girls to do well in school | 1 | 1 |
| Narrow careers paths are presented to teenage girls | 1 | 1 |
| Patriarchy creates pressure for girls in education, but not for boys | 1 | 1 |
| Patriarchy lifts boys up and they see their opportunities - not girls (education) | 1 | 1 |
| Potentially being limited by what they are taught in all girls school (skeptical) | 1 | 1 |
| Pressure on girls to do well and focus on education | 1 | 1 |
| Pressure to set yourself up to provide for yourself | 1 | 2 |
| Women have to try a lot harder than men | 1 | 1 |
| Women have to try a lot harder than men to be successful | 1 | 1 |
| Ramifications of 'failing' | 2 | 7 |
| Failing an exam as a girl is a risk to your later independence | 1 | 1 |
| Failing exams threatens girls' independence | 1 | 1 |
| Feeling you don't have second chances in education (girls in particular) | 1 | 1 |
| Girls face more education pressure because of implications | 1 | 1 |
| Girls failing exams means a lot more | 1 | 1 |
| Girls think they have limited opportunities - we should show them more | 1 | 1 |
| Not wanting to grow up and end up relying on a man | 1 | 1 |
| View of girls as more concerned about education and grades | 3 | 7 |
| Boys don't care as much about how smart other people are | 1 | 1 |
| Boys mess around in the classroom | 1 | 1 |
| For a boy, failing an exam isn't that bad | 1 | 1 |
| Girls are more anxious about academic achievement (and failure) | 1 | 1 |
| Girls are more focused on education | 1 | 1 |
| Girls are under more educational pressure and so are more likely to be anxious | 1 | 1 |
| Girls get really stressed about grades | 1 | 1 |
| c. Schools focus on the wrong thing | 6 | 103 |
| A need for flexiblity in school | 1 | 1 |
| Barriers to mental health and wellbeing support in school | 6 | 61 |
| Care with language - what to say and what not to say to children | 1 | 3 |
| Confidentiality is a barrier in the school context | 1 | 2 |
| Contradiction between schools talking about MH more & being biggest contributor | 1 | 1 |
| Dedicated staff at school are unapproachable | 1 | 2 |
| Feeling that taking space for mental health isn't accepted by school | 1 | 1 |
| It feels hard to talk to teachers about mental health | 1 | 1 |
| Lack of support leads to YP feeling issues aren't important | 1 | 1 |
| Lots of students don’t feel comfortable talking to teachers | 1 | 1 |
| Not comfortable talking to teachers because it’s too strict | 1 | 1 |
| Not enough time to focus on student happiness | 1 | 1 |
| Not every teacher or school staff is trusted to the same extent | 1 | 2 |
| Not everyone goes to talk to their teachers | 1 | 1 |
| Not everyone naturally feel comfortable talking about their issues - education important for those people | 1 | 1 |
| Nothing happens after sharing how you feel | 1 | 1 |
| One teacher being insensitive can cause wider mistrust | 1 | 1 |
| People can regret opening up if not handled well | 1 | 1 |
| Positive experience of speaking to adults at school about MH | 1 | 1 |
| Providing information of where to go for help is not enough | 1 | 1 |
| Providing support to students experiencing difficult circumstances | 1 | 1 |
| School counselling is too busy and people are turned away | 1 | 1 |
| School lacking in mental health support | 1 | 2 |
| School should do something after you share | 1 | 1 |
| Schools face time and resource issues in providing ongoing support | 1 | 1 |
| Schools need to acknowledge people’s mental health struggles | 1 | 1 |
| Schools need to be more relaxed with mental health | 1 | 1 |
| Schools need to stop making you feel unimportant or like there's nothing wrong with you | 1 | 1 |
| Schools should be more conscentious about challenges YP are experiencinhg | 1 | 3 |
| Schools should have considered their MH | 1 | 1 |
| Some school-based support is not professionally trained properly for mental health support | 1 | 1 |
| Some teachers signpost but don't listen | 1 | 2 |
| Some things schools do aren't that helpful | 1 | 1 |
| Teacher inflexibility in relation to mental health | 1 | 1 |
| Teachers and counsellors might expect you can handle and deal with things alone | 1 | 1 |
| Teachers aren't really 'there' when it comes to emotional support | 1 | 1 |
| Teachers aren't therapists - we can't expect them to fix this | 1 | 1 |
| Teachers currently do bare minimum in PSHE | 1 | 1 |
| Teachers doing a mindfulness break or opportunity to pause and be restful | 1 | 1 |
| Teachers don't always know what to say and do | 1 | 3 |
| Teachers need to understand what is BEHIND your needs | 1 | 1 |
| Teachers perceived as insensitive to mental health | 1 | 1 |
| Teachers share more personal experiences in college | 1 | 1 |
| Teachers should be more open about their personal experiences | 1 | 1 |
| Teachers should know what to do AFTER you talk | 1 | 1 |
| Teachers thinking sharing is enough | 1 | 1 |
| Teahcer responses could inadvertently make young people feel they aren't interested | 1 | 1 |
| Training for teachers needed | 1 | 1 |
| Trust issues around teachers and mental health | 1 | 1 |
| What happens AFTER you talk | 1 | 1 |
| Would be comfortable opening up to a teacher if school more relaxed | 1 | 1 |
| Would require confidentiality and discretion | 1 | 1 |
| Changing how we approach secondary education to be a more positive experience and period for growth | 1 | 1 |
| Could help students feel more comfortable going to school | 1 | 1 |
| Could improve attitudes about school generally | 1 | 1 |
| Dread school when it's too focused on wrong things | 1 | 1 |
| Feel like they don't have basic rights in school | 1 | 1 |
| Feeling unnecessarily penalized | 1 | 1 |
| Giving students more freedom will make students happier | 1 | 1 |
| More freedom and flexibility at college | 1 | 1 |
| More freedom led to enjoying school more | 1 | 1 |
| No freedom of expression in schools | 1 | 1 |
| School can be a hostile environment | 1 | 1 |
| School can be difficult if you are experiencing difficulties | 1 | 1 |
| Schools are too strict | 1 | 1 |
| Schools aren't inclusive for everyone | 1 | 1 |
| Schools being more relaxed would improve wellbeing | 1 | 1 |
| Schools do not allow diversity in expression | 1 | 1 |
| Schools don't have time for other things | 1 | 1 |
| Schools don’t make you feel heard | 1 | 1 |
| Schools focus on exams and dress code | 1 | 2 |
| Schools focus on the wrong things | 1 | 2 |
| Schools focused on deadlines | 1 | 1 |
| Schools focusing on the wrong thing means students don’t like school | 1 | 1 |
| Schools need to be more relaxed with uniform | 1 | 1 |
| Schools providing education on life issues | 1 | 1 |
| Schools should acknowledge different abilities | 1 | 1 |
| Schools should be less strict about deadlines | 1 | 2 |
| Schools should be more considerate | 1 | 2 |
| Schools should be more relaxed | 1 | 1 |
| Schools should think about what makes students happy | 1 | 1 |
| Schools take dress code too seriously | 1 | 1 |
| Sixth form is more relaxed than school | 1 | 1 |
| Some rules feel silly in school and this feeds inequality issues | 1 | 1 |
| Students behave when school is more relaxed | 1 | 1 |
| Teachers have a heavy emphasis on academic outcomes | 1 | 1 |
| Too focused on academic reputation | 1 | 1 |
| Toxic culture in studying fed by schools, colleges, and students | 1 | 1 |
| Young people come out of high school with negative mindsets that don't allow them to grow as people | 1 | 1 |
| D. Peer relationship difficulties | 7 | 131 |
| a. Judgement - comparison, competition, and conformity | 7 | 54 |
| A feeling of competition among teenage girls | 1 | 2 |
| Comparisons with friends can be upsetting | 1 | 1 |
| Comparisons with friends on looks, romantic experience | 1 | 1 |
| Competition & judgement around eating impacted self-esteem | 1 | 1 |
| Competition & pressure comes from ingrained prejudice towards women | 1 | 1 |
| Competition & pressure to be best ingrained from young age | 1 | 2 |
| Competition among girls to validate themselves and their feelings | 1 | 1 |
| Competition exists in all girls and mixed schools | 1 | 1 |
| Competition in girls to be the best is ingrained from society | 1 | 1 |
| Competition of who could eat the least (school) | 1 | 1 |
| Competition to get top grades | 1 | 1 |
| Constant competition makes it difficult to create own identity | 1 | 1 |
| Constantly comparing to others as a factor in self-esteem | 1 | 1 |
| Everything is a competition from young age | 1 | 1 |
| Expectation of conformity to friendship group | 1 | 1 |
| Expectations leads to pressure to conform | 1 | 1 |
| Fear of judgement comes from hearing other girls being judged | 1 | 1 |
| Feeling like other girls are talking behind your back | 1 | 1 |
| Feeling of competition = protection against being judged | 1 | 1 |
| Feeling you have to think like and agree with social media peers | 1 | 1 |
| Flex culture adds to pressure and feeling of competition | 1 | 1 |
| Friends internalise body norms and then encourage them with each other | 1 | 1 |
| Friendship difficulties based on boys and appearances are common | 1 | 1 |
| Girls have to figure out what has been said about them | 1 | 1 |
| Girls in constant competition with each other | 1 | 1 |
| Girls jealous of girls with eating disorders (school) | 1 | 1 |
| Girls judge each other on what they eat | 1 | 2 |
| Girls make snide comments about what other girls eat | 1 | 2 |
| Girls may project insecurities onto other girls based on what boys pervceive as attractive | 1 | 1 |
| Girls may project insecurities onto other girls based on what boys pervceive as attractive (2) | 1 | 1 |
| Girls subject to constant judgement | 1 | 1 |
| Insecurities forced on women by the media can cause competition and rivalry amongst women and girls | 1 | 1 |
| Jealousy and comparison around body image | 1 | 1 |
| Jealousy and comparison around what you plan to do with your life | 1 | 1 |
| Lots of jealousy and comparison for girls | 1 | 1 |
| Low self-esteem common in girls (lots of comparison to everyone) | 1 | 1 |
| Not doing enough compared to peers | 1 | 1 |
| Ostracized from friends for not achieving top grades | 1 | 1 |
| Peer pressure around body image, fed by social media | 1 | 1 |
| Peer pressure as a contributor | 1 | 1 |
| Pressure and stress to match status and fit | 1 | 1 |
| Pressure to be in a relationship | 1 | 1 |
| Pressure to conform to friends | 1 | 1 |
| Pressure to confrom & inability to be self impacts MH | 1 | 1 |
| Pressure to like the things and people your friends and peers like | 1 | 1 |
| Self-conscious that people are laughing at them (judging) | 1 | 1 |
| Status and feeling like you do or do not belong with friends | 1 | 1 |
| Teenage girls' insecurities create competition | 1 | 1 |
| Tendency to compare self to others | 1 | 1 |
| Want to skip lunchtime due to judgement from girls | 1 | 1 |
| b. Conflict | 7 | 64 |
| Also happens in online spaces | 4 | 10 |
| Backlash if you don't agree on social media | 1 | 1 |
| Bullied on social media | 1 | 1 |
| Bullying on social media is a negative aspect of social media | 1 | 1 |
| Cyberbullying and social media | 1 | 1 |
| Cyberbullying causes anxiety | 1 | 1 |
| Easy to see friends leaving you out on SM | 1 | 1 |
| Online communication can be misinterpreted | 1 | 2 |
| Social media can be used for bullying | 1 | 1 |
| Trolling and social media | 1 | 1 |
| Bullying | 2 | 35 |
| Being bullied impacts the person's identity;self-knowledge | 1 | 1 |
| Being bullied makes you feel bad about yourself | 1 | 1 |
| Being bullied makes you feel hopeless | 1 | 1 |
| Boys and girls bullied for talking to peers less popular than them | 1 | 1 |
| Boys are bullied to their faces; girls bullied behind their backs | 1 | 1 |
| Boys are bullied too | 1 | 3 |
| Bullying can occur from both boys and girls | 1 | 1 |
| Bullying contributes to low mood and anxiety | 1 | 1 |
| Bullying is more direct for boys | 1 | 1 |
| Bullying is more indirect for girls | 1 | 1 |
| Bullying towards girls tends to be indirect | 1 | 1 |
| Can be bullied for who you talk to | 1 | 1 |
| Difference in type of bullying from boys vs girls | 1 | 1 |
| Girls and boys are subject to different types of bullying | 1 | 1 |
| Girls and boys are subject to different types of bullying (2) | 1 | 1 |
| Girls can be mean to each other more indirectly | 1 | 1 |
| Girls can bully people as well as boys | 1 | 1 |
| Girls can get bullied for lots of reasons | 1 | 1 |
| Girls use indirect bullying | 1 | 1 |
| Girls' behaviour can feel like gaslighting | 1 | 1 |
| Got help for bullying and impact of bullying | 1 | 1 |
| Hard to compare who is bullied more | 1 | 1 |
| Indirect bullying from girls invalidates feelings and causes self-doubt | 1 | 1 |
| Indirect bullying impacts how you feel more | 1 | 1 |
| Lots of bullying between girls at high school | 1 | 1 |
| Not just feminine boys that are bullied | 1 | 1 |
| Some groups of girls can be nasty | 1 | 1 |
| Teenage girls can be horrible | 1 | 2 |
| Teenage girls can be horrible to each other | 1 | 1 |
| The teasing experienced by girls is different to that experienced by boys | 1 | 1 |
| Will be bullied by peers if girls don’t meet expectations | 1 | 1 |
| Worried she would always be bullied | 1 | 1 |
| Conflict and fallouts with friends and peers | 5 | 16 |
| Changes in communication methods during lockdown contributed to fall outs | 1 | 2 |
| COVID meant they couldn't escape school drama | 1 | 1 |
| Fall outs with friends impacts self-esteem | 1 | 1 |
| Friendship difficulties are common and challenging | 1 | 1 |
| Friendship issues don't only take place at school | 1 | 1 |
| Friendships are a big, big factor in distress | 1 | 1 |
| Friendships are a massive contributor to low mood | 1 | 1 |
| Misinterpretation of messages can cause fallouts | 1 | 1 |
| Misinterpretation of tone led to arguments | 1 | 1 |
| Peers as a contributing factor | 1 | 1 |
| Relationships and friendships main reason for upset in secondary school | 1 | 1 |
| Teenage girls trying to bring each other down | 1 | 1 |
| There isn't much we can do about how girls behave toward each other | 1 | 1 |
| Worry about being in the wrong click | 1 | 1 |
| You have clicks of girls at high school | 1 | 1 |
| Romantic breakups | 2 | 3 |
| Breakdown of romantic relationships contributes to low mood | 1 | 1 |
| Breakups and arguments and the fallout, can be upsetting | 1 | 1 |
| Breakups impact how you see yourself - damaging | 1 | 1 |
| c. Difficulties in distress among friends | 3 | 7 |
| Friends are going through the same thing so might not be able to be that helpful | 1 | 1 |
| Having friends that don't judge is necessary | 1 | 1 |
| If girls are increasingly eperiencing distress, are they affecting each other | 1 | 1 |
| Social and emotional contagion among girls | 1 | 1 |
| Stressful supporting and helping friends | 1 | 2 |
| Supporting friends having low mood and anxiety can affect you | 1 | 1 |
| d. Feeling isolated or alone | 2 | 6 |
| Being isolated at school is unbareable | 1 | 1 |
| Being unable to talk to friends made her feel lonely | 1 | 1 |
| Being unable to talk to friends made people lonely | 1 | 1 |
| Being unable to talk to friends was difficult | 1 | 1 |
| School is worst thing without friends | 1 | 1 |
| Try to avoid school when you have no friends there | 1 | 1 |
| **E. Social media as a space of comparison and insecurity** | 8 | 203 |
| a. Social media perpetuates expectations and encourages comparison and conformity | 8 | 69 |
| Comparison | 4 | 13 |
| Comparing self to other women on SM | 1 | 1 |
| Comparisons happen without thinking about it on social media | 1 | 1 |
| Constantly comparing self via social media | 1 | 1 |
| More social media comparisons in lockdown | 1 | 1 |
| Peer pressure stronger than influencers on SM | 1 | 1 |
| Social media adds to pressure to be better than everyone | 1 | 1 |
| Social media allows comparison to lots of people | 1 | 2 |
| Social media and comparisons | 1 | 3 |
| Social media makes you feel insecure | 1 | 1 |
| Young girls are comparing themselves to people on social media | 1 | 1 |
| Conformity and validation | 4 | 8 |
| Conformity, social media, and body image | 1 | 1 |
| encouraging social media users to think about why they are photoshopping their pictures could reduce the editing of photos | 1 | 1 |
| Feeling conflicted about wanting to be like someone online | 1 | 1 |
| Females looking for recognition and validation on SM | 1 | 1 |
| Pressure to conform so content is seen | 1 | 1 |
| Social media leading youth to conform (unconscious identity changes) | 1 | 1 |
| Trying to be like people you see online | 1 | 1 |
| Young women might be influenced to conform with favourite SM influencers | 1 | 1 |
| Intensity, repetition, pressure | 6 | 14 |
| it can feel like social media purposefully makes women feel insecure | 1 | 1 |
| Normalisation of insecurity on social media | 1 | 1 |
| Seeing an image once is okay | 1 | 1 |
| Seeing the same image repeatedly is harmful | 1 | 1 |
| Social media bring pressure | 1 | 1 |
| Social media creates unconscious expectations | 1 | 1 |
| Social media has subconscious effects | 1 | 1 |
| Social media makes you feel like you're not doing what you should | 1 | 1 |
| Social media puts pressure on girls to look a certain way | 1 | 1 |
| Social media showing you lots of body related points makes you question your own body | 1 | 1 |
| Social media subconsciously tells you how you should be | 1 | 1 |
| The biggest thing on social media is how you should look | 1 | 1 |
| TikTok algorithms become problematic - reiterate and pull you into body and image things | 1 | 2 |
| Sets standards | 5 | 10 |
| Beauty standards and social media | 1 | 1 |
| SM setting expectations in how you should look | 1 | 1 |
| Social media communicates body norms | 1 | 1 |
| social media forces teenage girls to play into the beauty standards it advertises | 1 | 3 |
| Social media girls should be thin and skinny | 1 | 1 |
| Social media has normalised very specific ways of looking to an extent that there is no real choice about how to look | 1 | 1 |
| Social media tells you to look a 'certain' way | 1 | 1 |
| Uniqueness is a good thing (but not portrayed this way on SM) | 1 | 1 |
| Unrealistic standards and difficulty telling what is 'real' | 4 | 24 |
| Beauty filters creating unrealistic expectations | 1 | 1 |
| Difficult for young people to recognise SM is a 'highlight reel' | 1 | 1 |
| Filters, photoshop, all create unrealistic standards | 1 | 1 |
| Increasing awareness that social media isn't real | 1 | 8 |
| Many girls are aware of editing social media photos but may need reminding that it's not real | 1 | 1 |
| Recognition that 'highlight reel' is only one aspect of their life | 1 | 1 |
| seeing inaccurate lifestyles on social media can be toxic | 1 | 1 |
| Show real selves to reduce unrealistic expectations | 1 | 1 |
| Showing real self will encourage others to | 1 | 1 |
| SM exposed you to people's 'highlight reel' | 1 | 1 |
| Social media and unrealistic beauty standards | 1 | 1 |
| Social media creates unrealistic standards | 1 | 1 |
| Social media filters are really realistic, so hard to know if they're used | 1 | 1 |
| Social media isn't always accurate | 1 | 1 |
| Social media portrays perfectness | 1 | 1 |
| Stop editing and using photoshop on pictures | 1 | 1 |
| Takes time to realise social media isn't real | 1 | 1 |
| b. How social media works, and how this feeds into issues (and how it could work better) | 8 | 83 |
| Problems are built into how social media works | 7 | 20 |
| (Social) media impact for women may not be deliberate, but is there nontheless | 1 | 1 |
| Algorithms on social media reduce your choice | 1 | 1 |
| Being realistic about what we can do about social media | 1 | 4 |
| Can't stop social media usage or movies being shown | 1 | 1 |
| it can feel like social media purposefully makes women feel insecure | 1 | 1 |
| Might be hard to identify accounts making you feel insecure | 1 | 1 |
| Pressure to conform so content is seen | 1 | 1 |
| Repeated imagery on multiple forms of medias – pictures and videos | 1 | 1 |
| Social media as absorbing time | 1 | 1 |
| Social media as addictive | 1 | 1 |
| Social media emphasis on how you look, act, behave - tiktok and insta | 1 | 1 |
| Social media filters are really realistic, so hard to know if they're used | 1 | 1 |
| social media forces teenage girls to play into the beauty standards it advertises | 1 | 3 |
| TikTok algorithms become problematic - reiterate and pull you into body and image things | 1 | 2 |
| Social media education and self-care needed (but autonomy alone isn't enough) | 3 | 19 |
| Delivering social media training | 1 | 1 |
| Education in social media provided by schools should be delivered at a much earlier age | 1 | 2 |
| Have to find the right accounts to follow on social media | 1 | 1 |
| If it's in users' hands to make changes, will they do it | 1 | 1 |
| Lessons about SM should cover bullying and self-image | 1 | 1 |
| Lessons on SM need to focus on current trends | 1 | 1 |
| Lessons on social media are outdated | 1 | 1 |
| Need to be taught about social media | 1 | 1 |
| Options exist to exert choice but people don't necessarily use them, need to be clearer and easier | 1 | 1 |
| People might not know that it's social media making them feel insecure | 1 | 1 |
| Providing support at the actual age when social media is first engaged with | 1 | 2 |
| Reduce time spent on phone | 1 | 1 |
| Social media training in primary school | 1 | 1 |
| Time limits on social media | 1 | 2 |
| Unfollowing accounts that make you feel insecure | 1 | 1 |
| Worth more effort from individual users to help curate more individually | 1 | 1 |
| The way we use social media | 6 | 11 |
| Family and parents don't understand phones and social media | 1 | 1 |
| Girls are accessing social media at younger ages | 1 | 1 |
| People are growing up with social media | 1 | 1 |
| People exposed to SM at younger ages (problem) | 1 | 1 |
| People spend hours on social media | 1 | 1 |
| Social media as a constancy | 1 | 1 |
| Social media more accessible to young people and children | 1 | 1 |
| Social media use increased in lockdown | 1 | 1 |
| Time on social media as without purpose | 1 | 1 |
| Widespread nature of social media - everyone uses it, lots of apps | 1 | 1 |
| Young people are even younger when they access phones and social media | 1 | 1 |
| Things that social media platforms could do | 3 | 25 |
| Changes to social media needed | 1 | 1 |
| Creators and social media companies need to be honest with their audience | 1 | 1 |
| Editing notices on social media posts could reduce the use of editing in their posts | 1 | 1 |
| Encourage engagement with support networks around social media | 1 | 2 |
| Enforce edit notifications just like paid promotions and fact checks | 1 | 2 |
| How would we regulate more disclosures of social media editing | 1 | 1 |
| More positive role models on social media needed | 1 | 2 |
| More regulations needed to protect young women on SM | 1 | 1 |
| More role models for girls (on social media) | 1 | 1 |
| Promotion of creators who are true self | 1 | 1 |
| Regulations needed more on instagram | 1 | 1 |
| Remove beauty filters | 1 | 2 |
| Skeptical that there could be regulation changes for SM | 1 | 1 |
| Social media companies should regulate age better | 1 | 1 |
| Social media needs to improve privacy settings | 1 | 1 |
| Social media should include notices that something is edited | 1 | 6 |
| Variation in what social media is | 2 | 4 |
| Different platform have different focuses | 1 | 1 |
| Different social media platforms bring different issues (some worse than others) | 1 | 1 |
| Insta = more editing of pictures | 1 | 1 |
| Instagram has greater impact on body image | 1 | 1 |
| Will be difficult to combat issues with social media now | 1 | 2 |
| You can't just eliminate everything on social media | 1 | 1 |
| You can't remove EVERYTHING but some control would be helpful | 1 | 1 |
| c. The impact of social media for self-concept and mental health | 7 | 29 |
| Early engagement with social media has lasting effects | 1 | 1 |
| Implications for mental health | 7 | 14 |
| Correlation between social media and increase in distress | 1 | 1 |
| Increase in rates links to increase in social media use | 1 | 1 |
| Low mood normal response to social media | 1 | 1 |
| Popularity of SM contributed to rates | 1 | 1 |
| Social media a huge part of the increase | 1 | 1 |
| Social media apps contributing to increase in poor mental health | 1 | 1 |
| Social media as reasoning behind increase | 1 | 1 |
| Social media contributes to low mood and anxiety | 1 | 1 |
| Social media contributes to rates | 1 | 2 |
| Social media impacting MH | 1 | 1 |
| Social media is the biggest contributor | 1 | 1 |
| Social media reduces mood | 1 | 1 |
| Trying to be like people online creates anxiety | 1 | 1 |
| Insecurity and self-esteem | 5 | 9 |
| (Social) media creates insecurities | 1 | 1 |
| Comparing to others lowers self-esteem (SM) | 1 | 1 |
| Comparing yourself to the images leads to insecurities in appearance | 1 | 1 |
| Images online influence a young person’s expectations of themselves | 1 | 1 |
| Instagram has greater impact on body image | 1 | 1 |
| SM impacts self-esteem | 1 | 1 |
| SM influences desire for plastic surgery | 1 | 1 |
| Social media and body image issues | 1 | 1 |
| Social media impacts girls self-esteem more than boys | 1 | 1 |
| Progression of negative effects from social media | 1 | 1 |
| Social media affects you subconsciously | 1 | 1 |
| Social media can be harmful | 1 | 2 |
| Social media is a toxic place | 1 | 1 |
| d. Caution in condemning social media | 7 | 22 |
| Anonymity of social media helps people talk about their MH | 1 | 1 |
| Different sides of social media - positive and negative | 1 | 1 |
| Easy to blame things on phones | 1 | 1 |
| Influencers speaking out could reduce stigma | 1 | 1 |
| Influencers speaking out will help normalise MH issues | 1 | 1 |
| It's not just about social media | 1 | 1 |
| More females are speaking out on SM | 1 | 1 |
| More openness and representation of women on SM | 1 | 2 |
| More people on SM expressing their sexuality | 1 | 1 |
| Negative connotations and judgement of social media - inherently gendered | 1 | 1 |
| School peers as well as beyond can be helpful to see their experiences, e.g., social media | 1 | 1 |
| SM can become a positive space for 'unheard' people | 1 | 1 |
| Social media brings awareness of social issues | 1 | 1 |
| Social media can be used to socialise but can be toxic | 1 | 1 |
| Social media can play both a positive and negative role | 1 | 1 |
| Social media has good and bad sides | 1 | 1 |
| Social media has positives and negatives | 1 | 1 |
| Social media helping people to be open about their MH | 1 | 1 |
| Social media helping to connect people with similar difficulties | 1 | 1 |
| Some social media has positivity - body and life positivity | 1 | 1 |
| Some social media platforms are harmless | 1 | 1 |
| **F. There is no easy answer** | 8 | 71 |
| a. Multiple causal routes all acting together | 4 | 16 |
| 'General' pressure on girls too young | 1 | 1 |
| Adolescence is a difficult time - developing identity | 1 | 1 |
| All kinds of contributing factors | 1 | 1 |
| Avoiding oversimplifying | 1 | 1 |
| Biological factors including hormones | 2 | 6 |
| Biological contributors - chemical imbalance | 1 | 1 |
| Biological contributors - hormones | 1 | 1 |
| Biological contributors - hormones (2) | 1 | 1 |
| Female puberty and hormonal changes not spoken about | 1 | 1 |
| Hormonal changes are confusing - no one speak about it | 1 | 1 |
| Puberty and hormones - 'changing in yourself' | 1 | 1 |
| General pressure on teenage girls - lots of sources | 1 | 1 |
| Lots of things building pressure for girls | 1 | 1 |
| Multiple contributors to difficulties | 1 | 1 |
| Not just one thing causing the increase | 1 | 1 |
| Several forms of pressure on girls is prevalent in society | 1 | 1 |
| Tackling one issue (or part) is enough | 1 | 1 |
| b. People are different | 7 | 38 |
| Intersectionality between gender and other identities | 2 | 8 |
| Lessons on gender and sexuality | 1 | 1 |
| Not their sexuality causing psych distress; its other people's reactions | 1 | 1 |
| Older generations’ reactions to people’s sexuality impact whether people come out | 1 | 1 |
| People struggle because of lack of sexuality conversations | 1 | 1 |
| Racism led to low self-esteem, low mood & anxiety | 1 | 1 |
| Scared about people’s reaction to their sexuality | 1 | 1 |
| Sexuality can contribute to girls’ low mood and anxiety | 1 | 1 |
| Sexuality not talked about enough in schools | 1 | 1 |
| People are different - and so are their difficulties and needs | 6 | 15 |
| 'Treatment' doesn't work for everyone | 1 | 1 |
| Coping = individual to the person (subjective) | 1 | 1 |
| Ensure diversity of people with lived experience | 1 | 1 |
| It can be hard to try to feel better | 1 | 1 |
| Opportunities to share feelings - including unconvential ones for those in need of it | 1 | 1 |
| people are different - not all affected the same | 1 | 1 |
| People are different and need different means of support | 1 | 1 |
| People's therapy and support needs may differ | 1 | 1 |
| Personal experiences can affect distress | 1 | 1 |
| Risk of stereotyping, is everyone the same | 1 | 1 |
| Training on different routes to suit different people's help-seeking interests | 1 | 1 |
| Trauma and abuse feeding into distress | 1 | 1 |
| Unable to engage with help for anxiety due to low mood | 1 | 1 |
| What works depends on the person | 1 | 1 |
| Youth with additional needs get frustrated with emotions | 1 | 1 |
| These things don't only affect teenage girls | 3 | 15 |
| Boys also develop body image issues (inc through social media) | 1 | 1 |
| Boys also experience expectation issues | 1 | 1 |
| Boys and girls both affected by school, but girls perhaps more | 1 | 1 |
| Boys are bullied if they present as feminine | 1 | 2 |
| Boys are bullied too | 1 | 3 |
| Boys might hide their femininity to avoid being bullied | 1 | 1 |
| Expectations affect girls and boys | 1 | 1 |
| Lockdown didn’t just impact teenage girls | 1 | 1 |
| Many people, not just teenage girls, felt lonely during lockdown | 1 | 1 |
| Not just feminine boys that are bullied | 1 | 1 |
| Pressures don't just affect girls | 1 | 1 |
| Shouldn't discount older people's MH issues | 1 | 1 |
| c. Change is hard (but worth it) | 6 | 17 |
| Awareness that we can't tackle every issue | 1 | 1 |
| Can't change everyone's beliefs | 1 | 1 |
| Can't change people's mindsets - limitation to action | 1 | 1 |
| Changes will lead to more changes | 1 | 1 |
| Damage already done prevents people from changing | 1 | 1 |
| Decision makers should be willing to make change | 1 | 1 |
| Dont know how we could help issues specific to girls | 1 | 2 |
| Issues around segregation - clashing needs | 1 | 1 |
| No change if people in pos. of power don't listen | 1 | 1 |
| Optimism that things can change | 1 | 1 |
| Spreading awareness is more realistic than outright solving the problem | 1 | 2 |
| Support and advice doesn't change your environment or challenges | 1 | 1 |
| These are big societal issues - how do we change societal thinking | 1 | 1 |
| What we should think about - it's difficult to overcome widespread societal issues, we can't eradicate inequality | 1 | 2 |
| ZZZ. Filtered out | 8 | 153 |
| 'Any step is a good step' | 1 | 1 |
| A need for further, ongoing support | 1 | 1 |
| Advice needed on how to remain independant in a relationship | 1 | 1 |
| Allowing mental health time away from school if needed | 1 | 1 |
| Avoiding algorithms and being able to exercise greater choice | 1 | 1 |
| Awareness of external factors | 1 | 1 |
| Being able to leave the house helped with impact of covid | 1 | 1 |
| Being open about feelings would help with impact of covid | 1 | 1 |
| Boys are physically bullied | 1 | 2 |
| Change from fuelling distress to helping | 1 | 1 |
| Compensating some expectations with other attributes | 1 | 1 |
| Creating ways to speak with other people | 1 | 1 |
| Current education on EDs not useful | 1 | 1 |
| Difficult to explain why women look for validation | 1 | 1 |
| Difficulty speaking to adults without feeling stupid | 1 | 1 |
| Distress can affect the future (exams, lifestyle) | 1 | 1 |
| Experiencing a MH issue forces you to learn about it | 1 | 1 |
| Facilitating autonomy in mental health and self-care | 1 | 1 |
| Family can be negative or a source of support for different people | 1 | 1 |
| Feeling singled out for support could reduce engagement | 1 | 1 |
| Feeling that mental health support services are not resourced enough | 1 | 1 |
| Friends struggle with eating at school too | 1 | 1 |
| Gain a better understanding when talking to people experiencing MH issues | 1 | 1 |
| Hard to talk to someone when you don’t know how they will react | 1 | 1 |
| Have lessons on MH and where to go for support | 1 | 1 |
| Having a whole class on MH in primary = helpful | 1 | 1 |
| Having space to share without judgement | 1 | 1 |
| Having specialised trained MH support in schools and colleges to help access | 1 | 1 |
| Help parents actually understand so they can help | 1 | 1 |
| Help parents manage their own emotional response | 1 | 1 |
| Help received not focused to both issues (anx & low mood) | 1 | 1 |
| Helping families understand how to help | 1 | 1 |
| Helping parents better understand what you are going through (and respond appropriately) | 1 | 1 |
| Helping parents understand complexities | 1 | 1 |
| Highlight reel leads to feeling anxiety you're behind everyone | 1 | 1 |
| If parents understand they can be a CHOICE of route | 1 | 1 |
| If parents understand they can help | 1 | 1 |
| Improve support in schools | 1 | 1 |
| Inability to acces help increases anxiety & low mood | 1 | 1 |
| Inability to access help may lead to serious, potentially fatal MH issues | 1 | 1 |
| Increase training for teachers | 1 | 1 |
| Increasing teacher understanding around low mood and anxiety specifically | 1 | 1 |
| Issues can present differently in girls | 1 | 1 |
| Key thing is that people find right support | 1 | 1 |
| Knowing there are support routes available is important | 1 | 1 |
| Lack of MH resourses leaves girls without support | 1 | 1 |
| Lack of school mental health support | 1 | 1 |
| Lack of support in schools normalising MH issues | 1 | 1 |
| Limited access to help makes MH issues worse | 1 | 1 |
| Long wait times for MH helplines | 1 | 1 |
| Look at statistics for hospital admissions | 1 | 1 |
| Look at statistics of referrals for CAMHS | 1 | 1 |
| Make school counselling more readily available | 1 | 1 |
| Make therapy and support embedded and accessible | 1 | 1 |
| Making sure people know they're not alone | 1 | 1 |
| Making sure when people ask for help, the response is right | 1 | 1 |
| Mental health is not tech company priority | 1 | 1 |
| Mental health resources and professionals should be more accessible | 1 | 2 |
| More option need to be available | 1 | 1 |
| Mental health support is not accessible | 1 | 1 |
| Mental health support requires sharing very personal things | 1 | 1 |
| Mentors beyond classroom as a good support routes | 1 | 1 |
| MH education helps to understand own symptoms | 1 | 1 |
| MH helplines need to be improved | 1 | 1 |
| Advice given by helpline volunteers isn't good | 1 | 1 |
| Call handlers need better training | 1 | 1 |
| Call handlers need more viable solutions | 1 | 1 |
| More places to direct people with less serious issues to | 1 | 1 |
| MH issues start when going into secondary school | 1 | 1 |
| MH needs to be spoken about earlier than secondary school | 1 | 1 |
| Provide information about MH services & resources | 1 | 1 |
| Secondary school is too late to start MH discussion | 1 | 1 |
| Speaking about MH earlier might be contraversial | 1 | 1 |
| Mistrust in MH services = do not seek help | 1 | 1 |
| More accessible help needed to catch things earlier | 1 | 1 |
| More people reaching out is a success | 1 | 1 |
| Multiple routes of signposting for different circumstances | 1 | 1 |
| Need to focus on prevention | 1 | 1 |
| Need to handle COVID better in future (schools) | 1 | 1 |
| Need to train more people who can provide help | 1 | 1 |
| Needing more choice about social media to avoid things that might affect you negatively | 1 | 2 |
| Needing people with knowledge and experience to support you | 1 | 2 |
| Not enough trained mental health professionals available | 1 | 1 |
| Not everyone might trust teachers ir feel cofortable sharing with them | 1 | 2 |
| Not penalizing prioritising your mental health | 1 | 1 |
| Other people feel the same but no way of knowing | 1 | 1 |
| Parent education doesn't need to place blame on parents | 1 | 1 |
| Parents as a barrier to the helpful action | 1 | 1 |
| Parents can simply be there for you, or they can listen if you open up | 1 | 1 |
| People in positions of influence should speak out about own MH issues | 1 | 1 |
| People with lived experience should teach about MH | 1 | 2 |
| Places you can turn to if you need help | 1 | 1 |
| Point of no return; prevention more feasible than a 'fix' | 1 | 1 |
| Positive role models exist but not enough of them | 1 | 1 |
| Prevention and early intervention (rather than treatment) is important | 1 | 1 |
| Prevention will help in the longer term | 1 | 1 |
| Professionals need to listen to people experiencing MH issues | 1 | 1 |
| Professionals working in MH should teach about it | 1 | 1 |
| Provide info on age appropriate MH services | 1 | 1 |
| Providing education will help identify MH issues earlier | 1 | 1 |
| Psychologically trained people | 1 | 1 |
| Refused help from CAMHS; not ill enough | 1 | 1 |
| Relied on friends to help each other through issues | 1 | 1 |
| Restrictions on getting further help | 1 | 1 |
| Restrictions on seeking help led to worsening MH & mistrust | 1 | 1 |
| Reuniting with family helped with the impact of covid | 1 | 1 |
| Same people who deliver teacher training should deliver parent training | 1 | 1 |
| Saying how you feel out loud is hard | 1 | 1 |
| School counsellors good for those with less support | 1 | 1 |
| School is a safe bet for support because you're there most of the time | 1 | 1 |
| School mental health discourses create stigma | 1 | 1 |
| School MH service patient evaluation forms ineffective | 1 | 1 |
| School professionals and families can all offer routes | 1 | 1 |
| School should have accessible MH support | 1 | 1 |
| School staff could be knowledgeable for support | 1 | 1 |
| School support might be more important for those who can't get help at home | 1 | 1 |
| Schools and young people could signpost parents to training | 1 | 1 |
| Service evaluation need to ask useful questions | 1 | 1 |
| Setting up a support network with multiple routes | 1 | 1 |
| Should learn about MH issues in PSHE | 1 | 2 |
| Should start to learn about MH in year 5 | 1 | 1 |
| Sometimes it's about just sharing - not looking for solutions | 1 | 2 |
| Sometimes talking without advice isn't that helpful | 1 | 1 |
| Space to talk through problems and share feelings | 1 | 2 |
| Start mental health and self-care conversations earlier | 1 | 1 |
| Support and help after someone shares issues | 1 | 1 |
| Support from professionals not accessible; too busy | 1 | 2 |
| Support on MH could improve wider parent-YP relationship | 1 | 1 |
| Support routes would improve mood and worry | 1 | 1 |
| Supporting a healthy balance between school work and friendships, community | 1 | 1 |
| Talk to someone to help with low mood and anxiety | 1 | 1 |
| Talking & educating youth about emotions from young age | 1 | 1 |
| Talking does help, but maybe not enough by itself | 1 | 1 |
| Teacher training on signs of distress | 1 | 1 |
| Teachers can inadvertently make things worse around mental health if not trained | 1 | 1 |
| Teachers COULD be a generally helpful route | 1 | 1 |
| Teaching on self-care | 1 | 1 |
| Teaching on supporting others | 1 | 1 |
| Tell parents it's not their fault | 1 | 1 |
| Therapy should be more available | 1 | 1 |
| To get indepth about MH need people with lived experience to teach | 1 | 1 |
| Trained organizations and groups should deliver training | 1 | 2 |
| Transition to secondary school a trigger for most people | 1 | 1 |
| Trust is important for sharing how you feel | 1 | 1 |
| Understanding of emotions help prevent worsening MH | 1 | 1 |
| Used to being around different types of girls | 1 | 1 |
| Vicious cycle of not understanding emotions leading to worsening | 1 | 1 |
| Waiting lists for therapy need to be sorted | 1 | 1 |
| Want trainers to be experienced and knowledgeable | 1 | 1 |
| Young people have favourite influencers online | 1 | 1 |
